# Rhubarb free anthraquinones improved mice nonalcoholic fatty liver disease by inhibiting NLRP3 inflammasome

**DOI:** 10.1186/s12967-022-03495-4

**Published:** 2022-06-28

**Authors:** Chao Wu, Yanqin Bian, Bingjie Lu, Dan Wang, Nisma Lena Bahaji Azami, Gang Wei, Feng Ma, Mingyu Sun

**Affiliations:** 1 Key Laboratory of Liver and Kidney Diseases (Ministry of Education), Institute of Liver Diseases, Shuguang Hospital, Shanghai University of Traditional Chinese Medicine, No.528 Zhangheng Road Pudong New District, Shanghai, 201203 China; 2 Shanghai University of Traditional Chinese Medicine, Shanghai, 201203 China; 3 Guanghua Hospital Affiliated to Shanghai University of Traditional Chinese Medicine, Shanghai, China; 4 State Key Laboratory of Genetic Engineering, Collaborative Innovation Center of Genetics and Development, School of Life Sciences, Human Phenome Institute, Fudan University, Shanghai, People’s Republic of China; 5 Suzhou Institute of Systems Medicine, Chinese Academy of Medical Sciences & Peking Union Medical College, Suzhou, 215123 People’s Republic of China

**Keywords:** Rhubarb free anthraquinones, NAFLD, NLRP3 inflammasome

## Abstract

**Background:**

Nonalcoholic fatty liver disease (NAFLD) is one of the most common chronic liver diseases and has become a huge public health issue worldwide. Inhibition of nucleotide oligomerization domain-like receptors containing pyrin domain 3 (NLRP3) inflammasome is a potential therapeutic strategy for NAFLD. Currently, there are no drugs targeting NLRP3 inflammasome for clinical treatment of NAFLD. In this study, we explored the efficacy and mechanism of rhubarb free anthraquinones (RFAs) in treating NAFLD by inhibiting NLRP3 inflammasome.

**Methods:**

First, NLRP3 inflammasome was established in mouse bone marrow-derived macrophages (BMDMs), Kuffer cells and primary hepatocytes stimulated by lipopolysaccharide (LPS) and inflammasome inducers to evaluate the effect of RFAs on inhibiting NLRP3 inflammasome and explore the possible mechanism. Further, Mice NAFLD were established by methionine and choline deficiency diet (MCD) to verify the effect of RFAs on ameliorating NAFLD by inhibiting NLRP3 inflammasome.

**Results:**

Our results demonstrated that RFAs including rhein/diacerein, emodin, aloe emodin and 1,8-dihydroxyanthraquinone inhibited interleukin-1 beta (IL-1β) but had no effect on tumor necrosis factor-alpha (TNF-α). Similar results were also showed in mouse primary hepatocytes and Kuffer cells. RFAs inhibited cleavage of caspase-1, formation of apoptosis-associated speck-like protein containing a CARD (ASC) speck, and the combination between NLRP3 and ASC. Moreover, RFAs improved liver function, serum inflammation, histopathological inflammation score and liver fibrosis.

**Conclusions:**

RFAs including rhein/diacerein, emodin, aloe emodin and 1,8-dihydroxyanthraquinone ameliorated NAFLD by inhibiting NLRP3 inflammasome. RFAs might be a potential therapeutic agent for NAFLD.

**Graphical Abstract:**

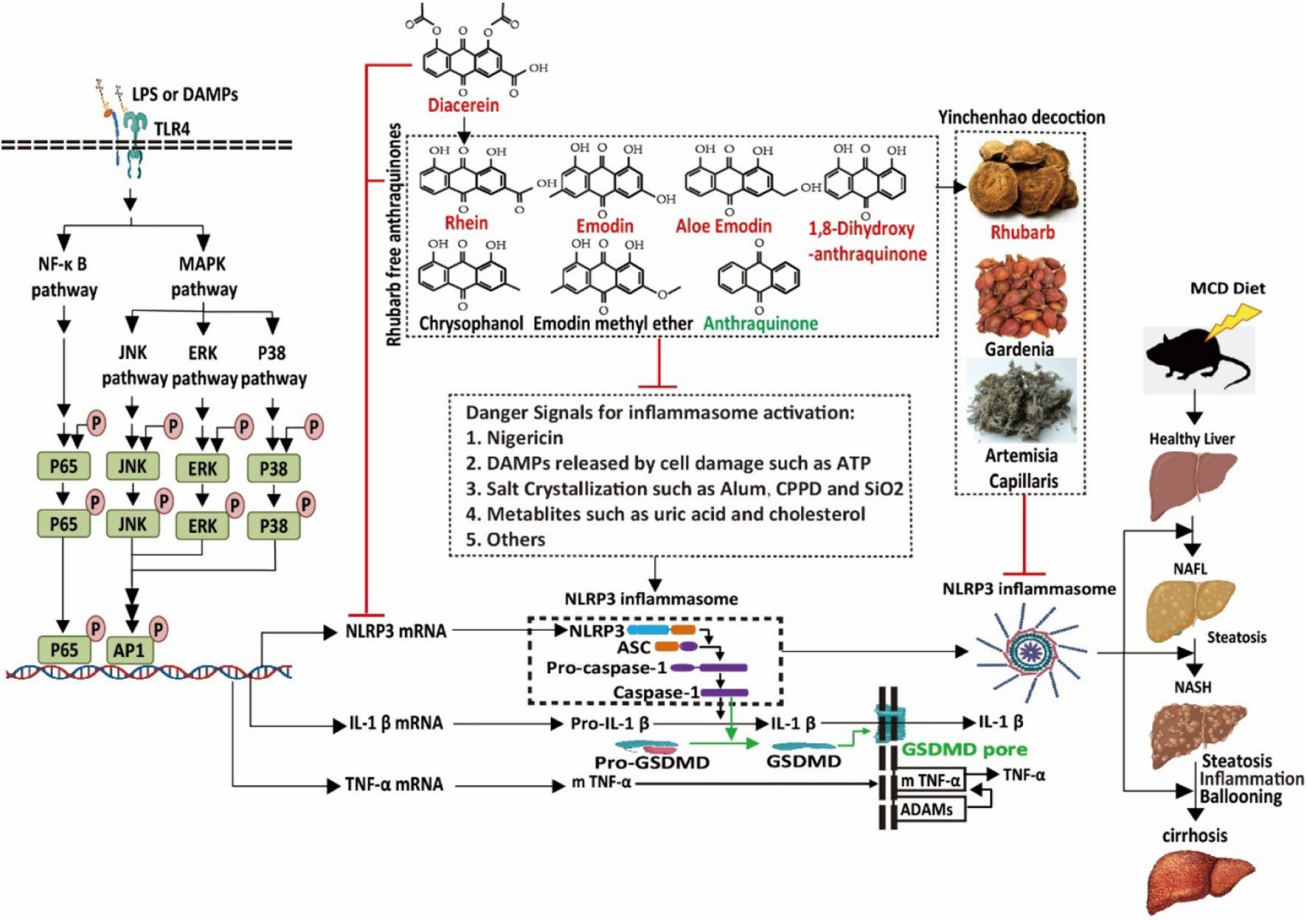

**Supplementary Information:**

The online version contains supplementary material available at 10.1186/s12967-022-03495-4.

## Highlights

1. Diacerein/rhein as NLRP3 blockers used in treating osteoarthritis clinically.
2. RFAs including rhein/diacerein are main components of Rhubarb contained in yinchenhao decoction.
3. RFAs inhibited the transcription and assembly of NLRP3 inflammasome.
4. RFAs, Rhubarb and yinchenhao decoction improved NAFLD probably by inhibiting NLRP3 inflammasome.
5. RFAs are the potential NLRP3 inflammasome blockers for treating NAFLD clinically.

**Supplementary Information:**

The online version contains supplementary material available at 10.1186/s12967-022-03495-4.

## Introduction

Nonalcoholic fatty liver disease (NAFLD) is characterized by steatosis in more than 5% of hepatocytes, which can be divided into simple steatosis, nonalcoholic steatohepatitis (NASH), cirrhosis and hepatocellular carcinoma according to the course of disease [1]. NAFLD affects approximately 25% of the global population and has become a huge public health issue [2]. NASH, as the progressive stage of NAFLD, contributes to the incidence of cirrhosis and hepatocellular carcinoma [3, 4]. Notably, NASH not only promotes the risk of type 2 diabetes mellitus, metabolic syndrome and cardiovascular events [4–6], but also increases the annual mortality rate of liver diseases and the all-cause mortality rate [4]. Although healthy eating and lifestyle, intestinal flora replacement and surgery have played a certain role in the prevention and treatment of NAFLD [7], the efficacy is not satisfactory due to the limitations of compliance, perseverance and tolerance, as well as surgery indications. Furthermore, weight control is the most important goals of these treatments, while about 40% of NAFLD patients are non-obese and nearly 25% of them are lean [8]. Recently, several drugs have entered different clinical trial stages: six compounds have completed phase 2 clinical trials and transferred to phase 3; dozens of additional treatment methods are in phase 2 clinical trials. Unfortunately, no clinical trials has shown that more than 50% of NAFLD patients achieve the primary treatment endpoint [9], so finding safe and effective drugs to ameliorate NAFLD remains an urgent clinical challenge.

Inflammasome activation in liver can lead to caspase-dependent cleavage of IL-1 and the pore-forming protein gasdermin D (GSDMD) which aggravates NFLAD through IL-1 induced inflammation and GSDMD mediated pyroptosis [10, 11]. First, NLRP3 inflammasome plays a vital role in the progression of NAFLD because it senses a wide range of danger signals related to metabolism such as cholesterol [10]. Secondly, NLRP3 knock in or knockout mouse provided important evidence about the role of NLRP3 inflammasome in the occurrence and development of NAFLD [12]. Finally, the number of discovered chemical compounds targeting inflammasomes increased, and animal experiments have been employed to assess the efficacy of these inflammasome inhibitors [13, 14], such as MCC950 and oridonin which showed the potential role in treating NAFLD [12, 15]. Although some inhibitors targeting NLRP3 inflammasome including MCC950 [16] and dapansutrile [17], and even some clinical drugs such as tranilast [18] and metformin [19], have shown regulatory effects on NLRP3 inflammasome, there are currently no drugs approved for clinical treatment of NFLAD.

Diacerein is a mature anti-inflammatory drug used in clinical treatment of osteoarthritis by inhibiting IL-1 through NLRP3 inflammasome, and rhein is the active product of diacerein [20, 21]. In addition, rhein is a vital component of rhubarb free anthraquinones (RFAs) which all share the similar molecular structure [22]. Therefore, we hypothesized that RFAs may prevent the progression of NAFLD by inhibiting NLRP3 inflammasome. Besides, we explored the effect of rhubarb and yinchenhao decoction (YCHD) on improving NAFLD by inhibiting NLRP3 inflammasome for the following reasons: (1) RFAs are the vital components of Chinese herb rhubarb (rheum palmatum L.) [22] which are used in many Chinese medicine formulas including rhubarb zhechong pill, YCHD, et al. for the treatment of liver diseases [23]; (2) YCHD is first recorded in *Shanghanlun* or *Treatise on Cold Damage Diseases* during the Eastern Han Dynasty and is beneficial to the treatment of liver diseases today because of its good and safe efficacy in clinical setting [24].

## Materials and methods

### Cell experiment

#### Isolation and culture of rat BMDMs

Mouse bone marrow cells were isolated from rat hind leg bone and differentiated (7 days) into macrophages in murine L929 media (diluted 1:10 in DMEM/RPMI with 10% FBS) within a humidified incubator containing 5% CO_2_ at 37 °C.

#### Isolation and culture of mouse primary hepatocytes and Kuffer cells

C57 mice were anesthetized with chloral hydrate. The abdominal cavity was opened and the portal vein was exposed. Portal vein puncture was performed with intravenous indwelling needle and the venous indwelling needle was fixed. 20 ml Hank’s solution (without Ca^2+^ and Mg^2+^) was used for portal vein perfusion for 5 min and the 50 ml Hank’s solution (with Ca^2+^, Mg^2+^, 1 mg/ml IV collagenase) was used for portal vein perfusion for 10–15 min. The liver was shredded, and 1640 medium was aspirated through a dropper to gently blow the liver tissue to separate single cell. The cell suspension was filtered with a 100mesh filter. The cell supernatant was then discared after centrifugation at 300 rpm for 5 min. (1) Isolation and culture of mouse primary hepatocytes: the cell precipitation was washed twice with 20 ml 1640 media and cell supernatant was discarded after centrifugation at 300 rpm for 5 min. Mouse hepatocytes were cultured in 1640 media with 10% FBS within a humidified incubator containing 5% CO_2_ at 37 °C for 2 h and then changed the media to remove the dead cell. Related experiments were carried out after 24–48 h of culture of adherent hepatocytes. (2) Isolation and culture of mouse Kuffer cells: the cell supernatant was centrifuged at 1500 rpm for 5 min, and the cell precipitation was collected for gradient centrifugation with percoll separation solution (up to down: 2 ml cell supernatant, 2 ml 25% percoll separation solution, 2 ml 50% percoll separation solution) at 2000*g* for 20 min. Kuffer cells were collected and cultured for 2 h in 1640 media with 10% FBS within a humidified incubator containing 5% CO_2_ at 37 °C for 2 h and then cleaned and changed. Related experiments were carried out when Kuffer cells ware cultured for 24–48 h. The Kuffer cells were identified by immunofluorescence through detecting F4/80 with the cell purity more than 90%.

#### Drug preparation

RFAs was dissolved in DMSO and filtered using a 0.2 μm cell filter. Artemisia capillaris granule (3.4 g), gardenia granules (1 g), rhubarb granules (1.4 g) were dissolved in 5 ml DMSO respectively and centrifuged at 1000 rpm/min for 15 min, and then the drug supernatant was filtered by 0.2 μm cell filter. YCHD: Artemisia capillaris granule (3.4 g), gardenia granules (1 g) and rhubarb granules (1.4 g) were dissolved in 5 ml DMSO together and centrifuged at 1000 rpm/min for 15 min, and then the drug supernatant was filtered by 0.2 μm cell filter.

#### Establishment of NLRP3 inflammasomes

Mouse BMDMs were activated by LPS (100 ng/ml) for 4 h and then stimulated by different inflammasome inducers: nigericin (2.5 μM) for 2 h, alum (100 μg/ml) for 12 h, SiO_2_ (100 μg/ml) for 12 h, CPPD (100 μg/ml) for 12 h, uric acid (100 μg/ml) for 12 h, cholesterol (100 μg/ml) for 12 h. Mouse primary hepatocytes were activated by LPS (1 μg/ml) for 4 h and then stimulated by cholesterol (100 μg/ml) for 12 h. Mouse Kuffer cells were activated by LPS (100 ng/ml) for 4 h and then stimulated by cholesterol (100 μg/ml) for 12 h.

#### Measurement of TLR4 pathway

Mouse BMDMs were activated by 100 ng/ml LPS for 0 min, 20 min and 40 min and then p-P65/P65, p-JNK/JNK, p-ERK/ERK and p-P38/p38 were detected by western blot.

#### Cell immunofluorescence for ASC specks detection

Mouse BMDMs were activated by 100 ng/ml LPS for 4 h and then then stimulated by nigericin (2.5 μM) for 2 h with or without the change of LPS-stimulated cell culture media. Mouse Kuffer cells were activated by LPS (100 ng/ml) for 4 h and then stimulated by cholesterol (100 μg/ml) for 12 h. The cells were fixed with 4% paraformaldehyde fix solution for 1 h and then blocked with 5% bovine serum albumin (BSA) solution for 1 h. The cells were incubated with ASC antibody for 24 h and then incubated with anti-rabbit IgG-Cy3 for 2 h. The nuclei were stained with 4′,6-diamidino-2-phenylindole (DAPI) for 15 min.

#### RT-qPCR

RNA was isolated by RNAfast200 kit. Reverse transcription and amplification were performed with the TAKARA reverse transcription kit and Toyobo amplification kit. Mouse primers provided as follows: 5′-AGTGTGACGTTGACATCCGT-3′ (sense) and 5′-GCAGCTCAGTAACAGTCCGC-3′ (antisense) for β-actin; 5′-TGGATGGGTTTGCTGGGAT-3′ (sense) and 5′-CTGCGTGTAGCGACTGTTGAG-3′ (antisense) for NLRP3.

#### Western blot

Collected samples were first electrophoresed at 80 V, then at 120 V. The condition of protein transmembrane was 18 V for 2.5 h. The first antibody was incubated at 4 ℃ overnight, and the second antibody was incubated at room temperature for 2 h.

#### Measurements of factors in cell culture supernatant

Lactate Dehydrogenase (LDH) was measured using LDH kit. IL-1β and TNF-α in supernatant were measured using IL-1β/TNF-α ELISA kits.

### Animal experiment

#### Experimental process

(A) 65 C57BL/6 mice were randomly divided into 13 groups (n = 5/group): control group was given methionine and choline supplemented (MCS) diet, model group was given methionine and Choline deficient (MCD) diet. The treatment groups were given MCD diet and different drugs including YCHD, artemisia capillaris, gardenia, rhubarb, rhein, diacerin, emodin, aloe emodin, 1,8-dihydroxyanthraquinone, MCC950 and VX765. This experiment lasted for 3 weeks and the mice were sacrificed by carbon dioxide anesthesia to obtain serum and liver tissue. (B) 96 C57BL mice were randomly divided into 13 groups: control group (n = 5/group) was given MCS diet, model group (n = 14/group) was given MCD diet. The treatment groups (n = 7/group) were given MCD diet and different drugs including YCHD, artemisia capillaris, gardenia, rhubarb, rhein, diacerein, emodin, aloe emodin, 1,8-dihydroxyanthraquinone, MCC950 and VX765. This experiment lasted for 9 weeks and the mice were sacrificed by carbon dioxide anesthesia to obtain serum and liver tissue. All C57BL male mice (20 ± 2 g, SPF grade) were provided by the Experimental Animal Center of Fudan University and Experimental Animal Center of Shanghai University of traditional Chinese Medicine. All mice were maintained at an animal facility under pathogen-free conditions. The handling of mice and experimental procedures were conducted in accordance with experimental animal guidelines. This study was approved by the laboratory animal ethics committee of Fudan University: 2019–02-SGYY-SMY-01 as well as Shanghai University of traditional Chinese Medicine: PZSHUTCM201820005.

#### Drugs intervention

Artemisia capillaris granule (3.4 g), gardenia granules (1 g), rhubarb granules (1.4 g) rhubarb (15 mg), rhein (15 mg), diacerin (15 mg), emodin (15 mg), aloe emodin (15 mg), 1,8-dihydroxyanthraquinone (15 mg), MCC950 (10 mg) and VX765 (10 mg) were dissolved in 10 ml 0.4% sodium carboxymethyl cellulose solution respectively. YCHD is the mixture of artemisia capillaris granule (3.4 g), gardenia granules (1 g), rhubarb granules (1.4 g) which were dissolved in 10 ml 0.4% sodium carboxymethyl cellulose solution together. All drugs were given by gavage every 2 days.

#### Hematoxylin and eosin (H & E) stain and sirius red staining

The largest lobe of liver was fixed in 4% paraformaldehyde and paraffin embedded, then 4 mm thick sections were cut and stained with H&E stain as well as sirius red staining.

#### Measurements of factors in serum

Alanine aminotransferase (ALT) and aspartate aminotransferase (AST) were measured using ALT/AST kit. Serum IL-1β and TNF-α were measured using IL-1β/TNF-α ELISA kits.

## Results

### RFAs inhibited NLRP3 inflammasome

**Fig. 1:**
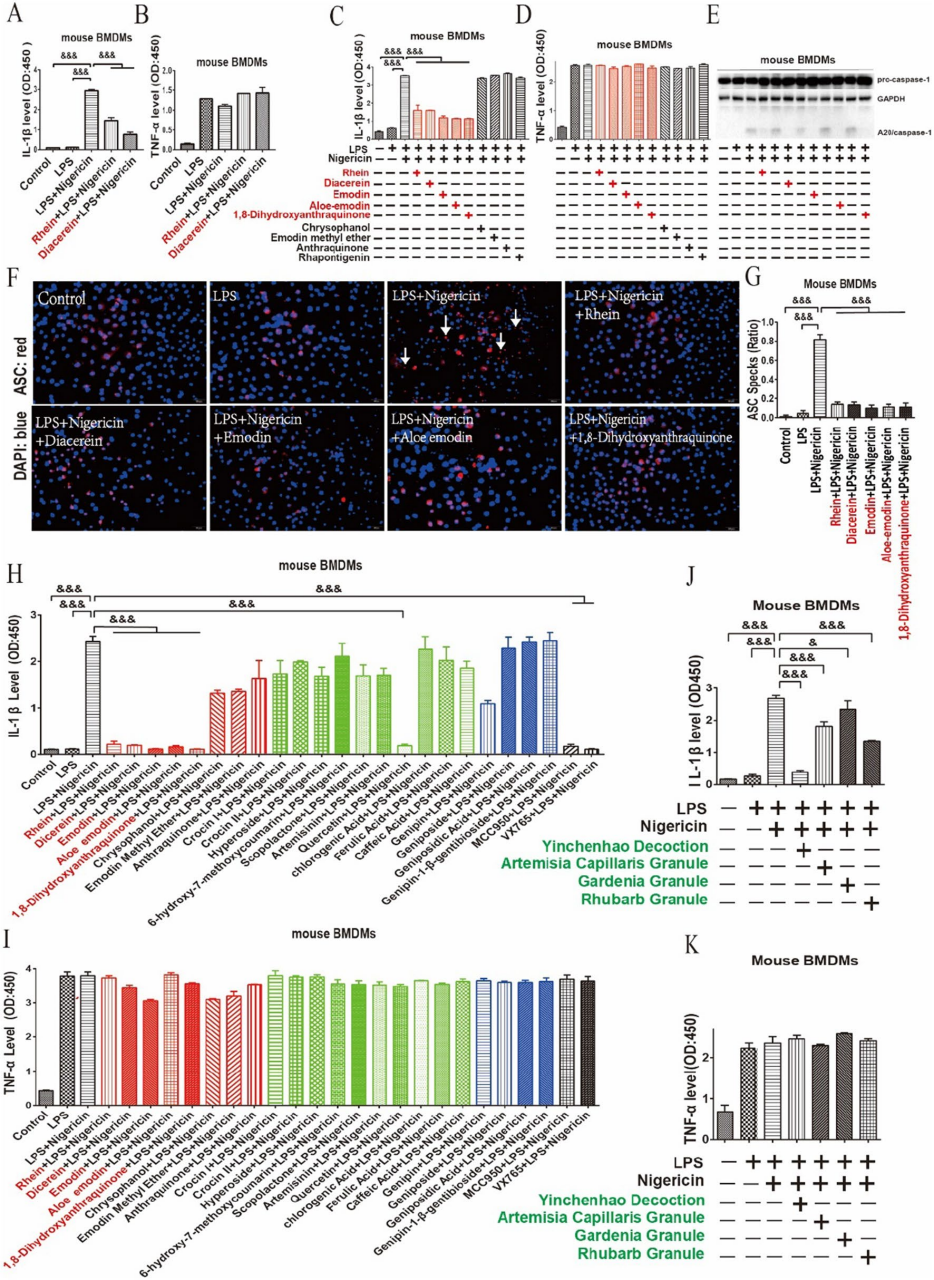
RFAs inhibited NLRP3 inflammasome. Mouse BMDMs were pretreated by diacerein (40 μM) and rhein (40 μM) for 30 min and then stimulated with LPS (100 ng/ml) for 4 h and nigericin (2.5 μM) for 2 h. IL-1β (**A**) and TNF-α (**B**) in cell culture supernatant were detected by ELISA (n = 3/group). Mouse BMDMs were pretreated by RFAs (40 μM rhein/40 μM diacerein, 40 μM emodin, 40 μM aloe emodin, 40 μM 1,8-dihydroxyanthraquinone, 20 μM chrysophanol, 4 μM emodin methyl ether, 4 μM anthraquinone) for 30 min and then stimulated with LPS (100 ng/ml) for 4 h and nigericin (2.5 μM) for 2 h. IL-1β (**C**) and TNF-α (**D**) in cell culture supernatant were detected by ELISA (n = 3/group), the cleavage of procaspase-1 (**E**) was detected by western blot, the ASC specks (**F**, **G**) were detected by immunofluorescence. Mouse BMDMs were pretreated with some important pharmaceutical ingredients derived from artemisia capillaris (green columns), gardenia (blue columns) and rhubarb (red columns) for 30 min and then stimulated with LPS (100 ng/ml) for 4 h. Nigericin (2.5 μM) was added for 2 h. IL-1β (**H**) and TNF-α (**I**) in cell culture supernatant were detected by ELISA (n = 3/group). Mouse BMDMs were pretreated by YCHT (5 μl/ml), artemisia capillaris (5 μl/ml), gardenia (5 μl/ml) and rhubarb (5 μl/ml) for 30 min and then stimulated with LPS (100 ng/ml) for 4 h and nigericin (2.5 μM) for 2 h. IL-1β (**J**) and TNF-α (**K**) in cell culture supernatant were detected by ELISA (n = 3/group). Data are presented as mean ± SEM. For multiple comparisons, one-way ANOVA coupled with LSD’s post hoc testing was performed. &: p < 0.05; &&: p < 0.01; &&&: p < 0.001. ANOVA, analysis of variance; ASC, apoptosis-associated speck-like protein containing CARD; BMDMs, bone marrow-derived macrophages; DAPI, 4′,6-diamidino-2-phenylindole; ELISA, enzyme linked immunosorbent assay; IL-1β, interleukin-1 beta; LPS, lipopolysaccharide; TNF-α, tumor necrosis factor-alpha

Diacerein is a mature anti-inflammatory drug by inhibiting NLRP3 inflammasome and rhein is the active product of diacerein [20, 21]. Here we verified this result in mouse BMDMs stimulated by LPS + nigericin which activates NLRP3 inflammasome [25]. Rhein and diacerein inhibited IL-1β (Fig. 1A), but had no effect on TNF-α (Fig. 1B). Furthermore, rhein is a vital component of RFAs which all have the similar molecular structure [22]. Therefore, we explored the role of these RFAs in inhibiting NLRP3 inflammasome. RFAs including rhein/diacerein, emodin, aloe emodin and 1,8-dihydroxyanthraquinone inhibited NLRP3 inflammasome by inhibiting IL-1β (Fig. 1C), the cleavage of pro-caspase-1(Fig. 1E) and the formation of ASC specks (Fig. 1F, G) in mouse BMDMs stimulated by LPS + nigericin, but bad no effect on TNF-α (Fig. 1D). NLRP3 inflammasome are also activated by LPS + alum/SiO_2_/CPPD/uric acid to induce NLRP3 inflammasome [26–29], while these RFAs inhibited these inducers activated NLRP3 inflammasome by inhibiting IL-1β (Additional file 1: Fig. S1A), but had no effect on TNF-α (Additional file 1: Fig. S2B). These RFAs inhibited the IL-1β (Additional file 1: Fig. S2A), LDH (Additional file 1: Fig. S2B) and the cleavage of pro-caspase-1 (Additional file 1: Fig. S2C) in a dose dependent manner in mouse BMDMs stimulated by LPS + nigericin.

RFAs are the vital components of herb rhubarb used in many Chinese medicine formulas for the treatment of liver diseases including YCHD composed of artemisia capillaris, gardenia and rhubarb [22, 24]. Therefore, we explored the role of YCHD, hers and some components of YCHD in inhibiting NLRP3 inflammasome. RFAs including rhein/diacerein, emodin, aloe emodin and 1,8-dihydroxyanthraquinone were the vital components of rhubarb, rhubarb and YCHD to inhibit NLRP3 inflammasome by inhibiting IL-1β (Fig. 1H, J), but had no effect on TNF-α (Fig. 1I, K).

### RFAs inhibited the transcription and assembly of NLRP3

**Fig. 2:**
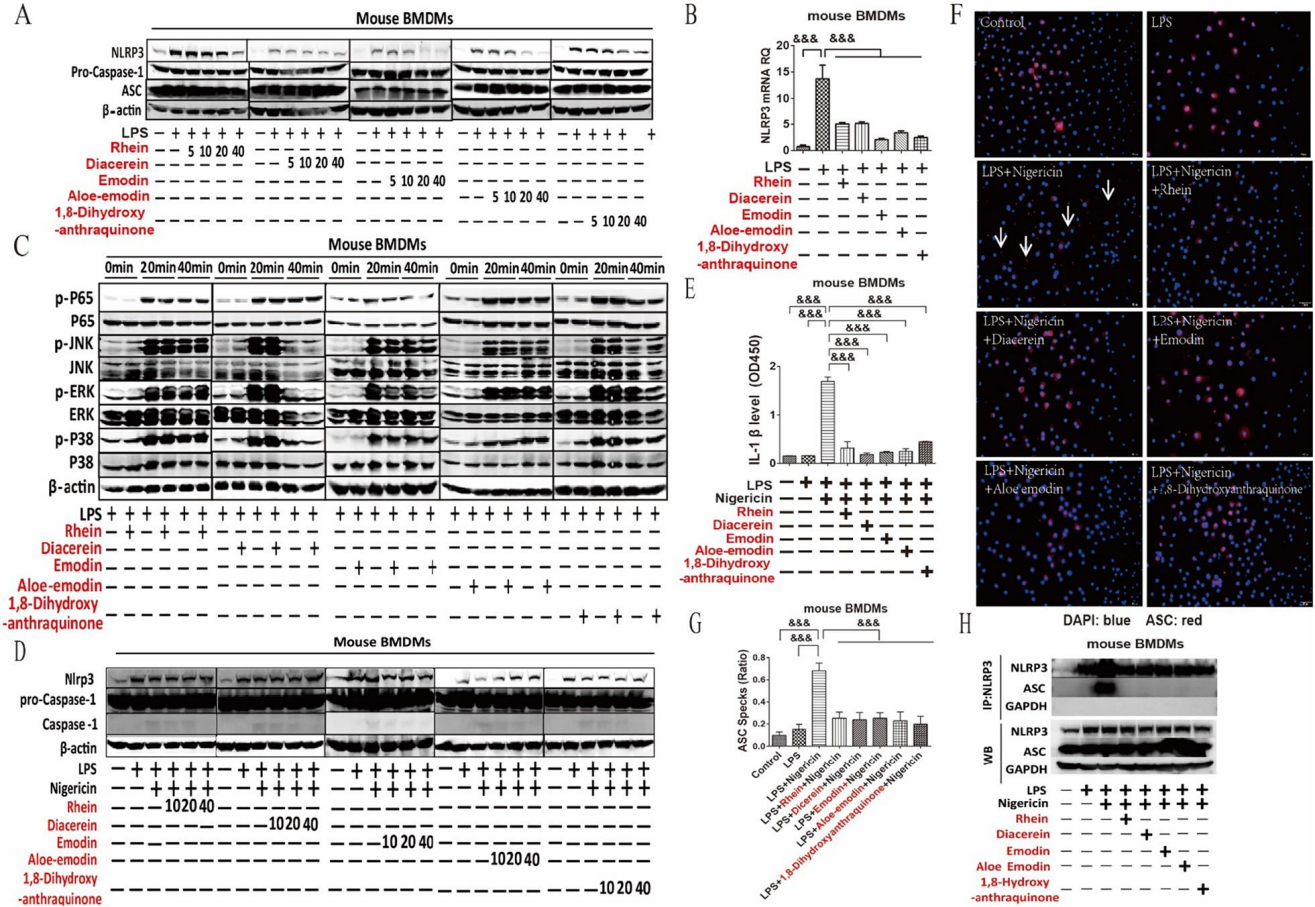
RFAs inhibited both the transcription and assembly of NLRP3 inflammasome. Mouse BMDMs were pretreated by rhein, diacerein, emodin, aloe emodin and 1,8-dihydroxyanthraquinone with the concentrations of 40 μM, 20 μM, 10 μM, and 5 μM for 30 min and then stimulated with LPS (100 ng/ml) for 4 h. NLRP3, ASC and pro-caspase-1 (**A**) were detected by western blot. Moue BMDMs were pretreated with rhein, diacerein, emodin, aloe emodin, 1,8-dihydroxyanthraquinone (all 40 μM) for 30 min and then stimulated with LPS (100 ng/ml) for 1 h. NLRP3 mRNA (**B**) was detected by RT-qPCR (n = 3/group). Mouse BMDMs were pretreated by rhein, diacerein, emodin, aloe emodin and 1,8-dihydroxyanthraquinone (all 40 μM) for 30 min and then stimulated with LPS (100 ng/ml) for 0 min, 20 min and 40 min. The phosphorylation of P65, P38, JNK and ERK (**C**) were detected by western blot. Mouse BMDMs were stimulated by LPS (100 ng/ml) for 4 h and then changed the media. Rhein, diacerein, emodin, aloe emodin and 1,8-dihydroxyanthraquinone with the concentrations of 40 μM, 20 μM, 10 μM, and 5 μM were added for 30 min and then the BMDMs were stimulated with nigericin (2.5 μM) for 2 h. The protein expression of NLRP3, ASC and pro-caspase-1 as well as the cleavage of pro-caspase-1 (**D**) were detected by western blot. Mouse BMDMs were stimulated by LPS (100 ng/ml) for 4 h before the media was changed. Rhein, diacerein, emodin, aloe emodin and 1,8-dihydroxyanthraquinone (all 40 μM) were added for 30 min and then stimulated with nigericin (2.5 μM) for 2 h. IL-1β (**E**) in cell culture supernatant was detected by ELISA (n = 3/group); The ASC specks (**F**, **G**) was detected by immunofluorescence; the combination of NLRP3 and ASC (**H**) was detected by immunoprecipitation. Data are presented as mean ± SEM. For multiple comparisons, one-way ANOVA coupled with LSD’s post hoc testing was performed. &: p < 0.05; &&: p < 0.01; &&&: p < 0.001. ANOVA, analysis of variance; ASC, apoptosis-associated speck-like protein containing CARD; BMDMs, bone marrow-derived macrophages; DAPI, 4',6-diamidino-2-phenylindole; ELISA, enzyme linked immunosorbent assay; IL-1β, interleukin-1 beta; LPS, lipopolysaccharide; RT-qPCR, quantitative reverse transcription polymerase chain reaction; TNF-α, tumor necrosis factor-alpha

The effect of rhein, diacerein, emodin, aloe emodin, and 1,8-dihydroxyanthraquinone on the transcription of NLRP3 was explored. Effective RFAs significantly inhibited NLRP3 protein expression (Fig. 2A) in a dose dependent manner as well as the NLRP3 mRNA expression in mouse BMDMs stimulated by LPS (Fig. 2B, Additional file 1: Fig. S3). LPS-activated TLR4 pathway including nuclear factor kappa-B (NF-κ B) pathway and mitogen-activated protein kinase (MAPK) pathway were associated with the transcription of NLRP3 [30, 31]. While these effective RFAs had little effect on both NF-κ B pathway and MAPK pathway with little effect on the phosphorylation of P65, JNK, ERK and P38 (Fig. 2C). Overall, rhein, diacerein, emodin, aloe emodin, and 1,8-dihydroxyanthraquinone inhibited the NLRP3 transcription independent of TLR4 signaling pathway.

The direct role of rhein, diacerein, emodin, aloe emodin, and 1,8-dihydroxyanthraquinone in regulating the assembly of NLRP3 inflammasome was explored by changing cell culture medium with LPS before the treatment of these abovementioned anthraquinones and the stimulation of nigericin. All these effective RFAs inhibited the cleavage of pro-caspase-1 in a dose dependent manner but had no effect on NLRP3 protein expression (Fig. 2D). All these effective RFAs inhibited the secretion of IL-1β in a dose-depend manner (Fig. 2E, Additional file 1: Fig. S4). These effective RFAs also inhibited the formation of ASC specks (Fig. 2F, G) and the protein binding between NLRP3 and ASC (Fig. 2H). These results showed that these RFAs directly inhibited the assembly of NLRP3 inflammasome.

### RFAs inhibited cholesterol induced NLRP3 inflammasome

**Fig. 3:**
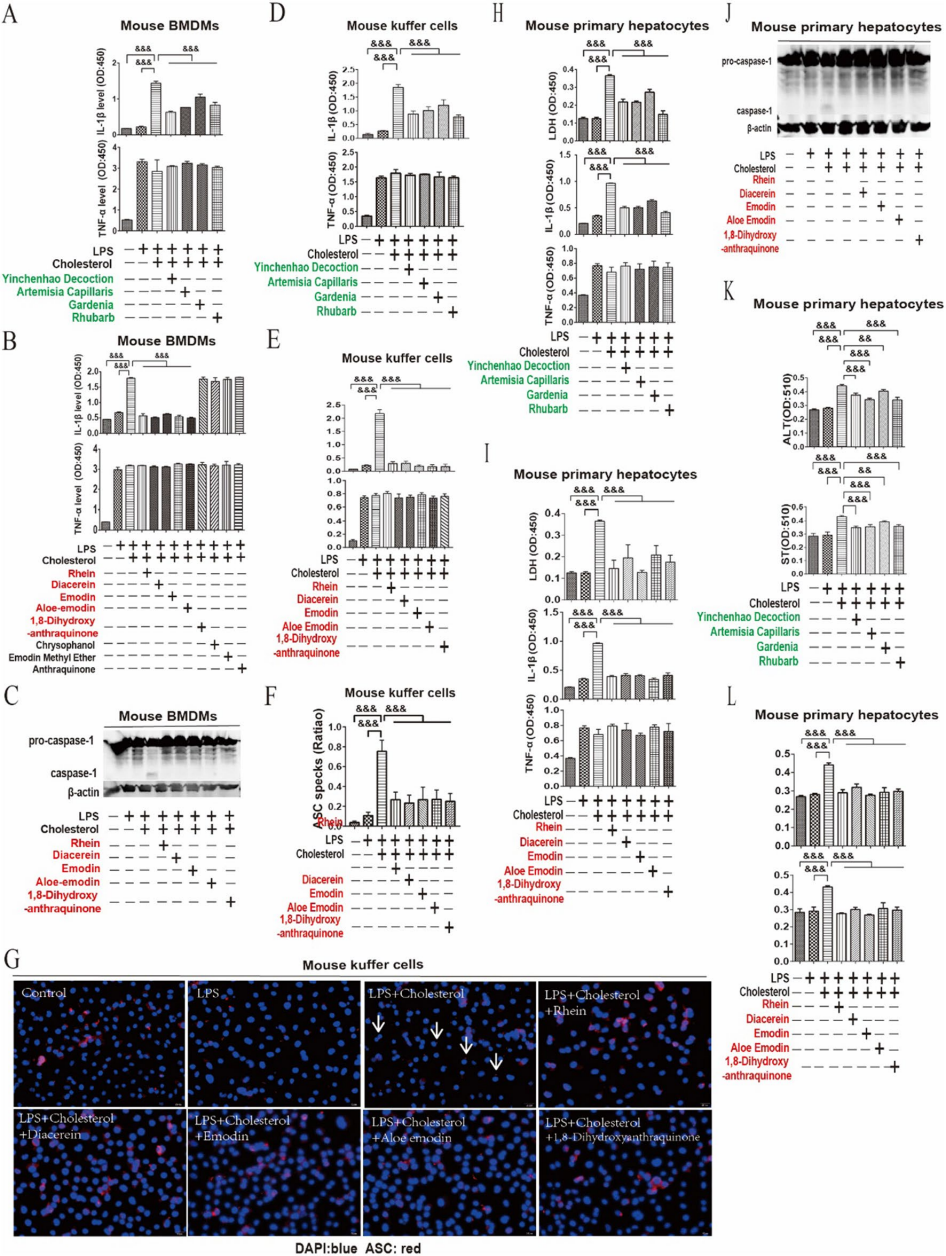
RFAs inhibited cholesterol induced NLRP3 inflammasome. Mouse BMDMs and Kuffer cells were pretreated with YCHT (5 μl/ml), artemisia capillaris (5 μl/ml), gardenia (5 μl/ml) and rhubarb (5 μl/ml) as well as RFAs (rhein/diacerein, emodin, aloe emodin, 1,8-dihydroxyanthraquinone, all 40 μM) for 30 min and then stimulated with LPS (100 ng/ml) for 4 h and cholesterol (100 μg/ml) for 12 h. In mouse BMDMs, IL-1β and TNF-α in cell culture supernatant were detected by ELISA (**A**,** B**) (n = 3/group), and the cleavage of procaspase-1influced by RFAs was detected by western blot (**C**). In mouse Kuffer cells, IL-1β and TNF-α in cell culture supernatant were detected by ELISA (**D**,** E**) (n = 3/group), and the ASC specks influenced by RFAs were detected by immunofluorescence (**F**, **G**). Mouse primary hepatocytes were pretreated with YCHT (5 μl/ml), artemisia capillaris (5 μl/ml), gardenia (5 μl/ml) and rhubarb (5 μl/ml) as well as RFAs (rhein/diacerein, emodin, aloe emodin, 1,8-dihydroxyanthraquinone, all 40 μM) for 30 min and then stimulated with LPS (1 μg/ml) for 4 h and cholesterol (100 μg/ml) for 12 h. IL-1β and TNF-α in cell culture supernatant were detected by ELISA and LDH were detected by LDH kits (**H**,** I**), ALT and AST were detected by ALT/AST kits (**K**,** L**), and the cleavage of procaspase-1 influenced by RFAs was detected by western blot (**J**). Data are presented as mean ± SEM. For multiple comparisons, one-way ANOVA coupled with LSD’s post hoc testing was performed. &: p < 0.05; &&: p < 0.01; &&&: p < 0.001. ANOVA; analysis of variance; ALT, alanine aminotransferase; ASC, apoptosis-associated speck-like protein containing CARD; AST, aspartate aminotransferase; BMDMs, bone marrow-derived macrophages; DAPI, 4′,6-diamidino-2-phenylindole; ELISA, enzyme linked immunosorbent assay; IL-1β, interleukin-1 beta; LDH, lactate dehydrogenase; LPS, lipopolysaccharide; TNF-α, tumor necrosis factor-alpha

Metabolites such as cholesterol can activate NLRP3 inflammasome in the pathological process of NAFLD [32]. Therefore, the role of RFAs including rhein/diacerein, emodin, aloe emodin and 1,8-dihydroxyanthraquinone, rhubarb and YCHT in regulating NLRP3 inflammasome was explored in mouse BMDMs, Kuffer cells and primary hepatocytes stimulated by LPS + cholesterol. In mouse BMDMs, these effective RFAs, rhubarb and YCHD inhibited IL-1β (Fig. 3A, B, Additional file 1: Fig. S5A), but had no effect on TNF-α (Fig. 3A, B, Additional file 1: Fig. S5B); besides, these effective RFAs also inhibited the cleavage of pro-caspase-1 (Fig. 3C). Mouse Kuffer cells were identified by immunofluorescence through detecting F4/80 (Additional file 1: Fig. S6A). In mouse Kuffer cells, these effective RFAs, rhubarb and YCHD inhibited IL-1β (Fig. 3D, E, Additional file 1: Fig. S6B), but had no effect on TNF-α (Fig. 3D, E, Additional file 1: Fig. S6C); besides, these effective RFAs also inhibited the formation of ASC specks (Fig. 3F, G). In mouse primary hepatocytes, cholesterol resulted in the increase of LDH (Additional file 1: Fig. S7A), IL-1β (Additional file 1: Fig. S7B), TNF-α (Additional file 1: Fig. S7C), ALT (Additional file 1: Fig. S8A) and AST (Additional file 1: Fig. S8B), while these effective RFAs, rhubarb and YCHD inhibited the LDH (Fig. 3H, I, Additional file 1: Fig. S7D), IL-1β (Fig. 3H, I, Additional file 1: Fig. S7E), ALT (Fig. 3K, L, Additional file 1: Fig. S8C) and AST, (Fig. 3K , L, Additional file 1: Fig. S8D), but had no effect on TNF-α (Fig. 3H, I, Additional file 1: Fig. S7F). Besides, these effective RFAs also inhibited the cleavage of pro-caspase-1 (Fig. 3J) in mouse primary hepatocytes.

### RFAs improved MCD diet induced mice NAFLD by inhibiting NLRP3 inflammasome

**Fig. 4:**
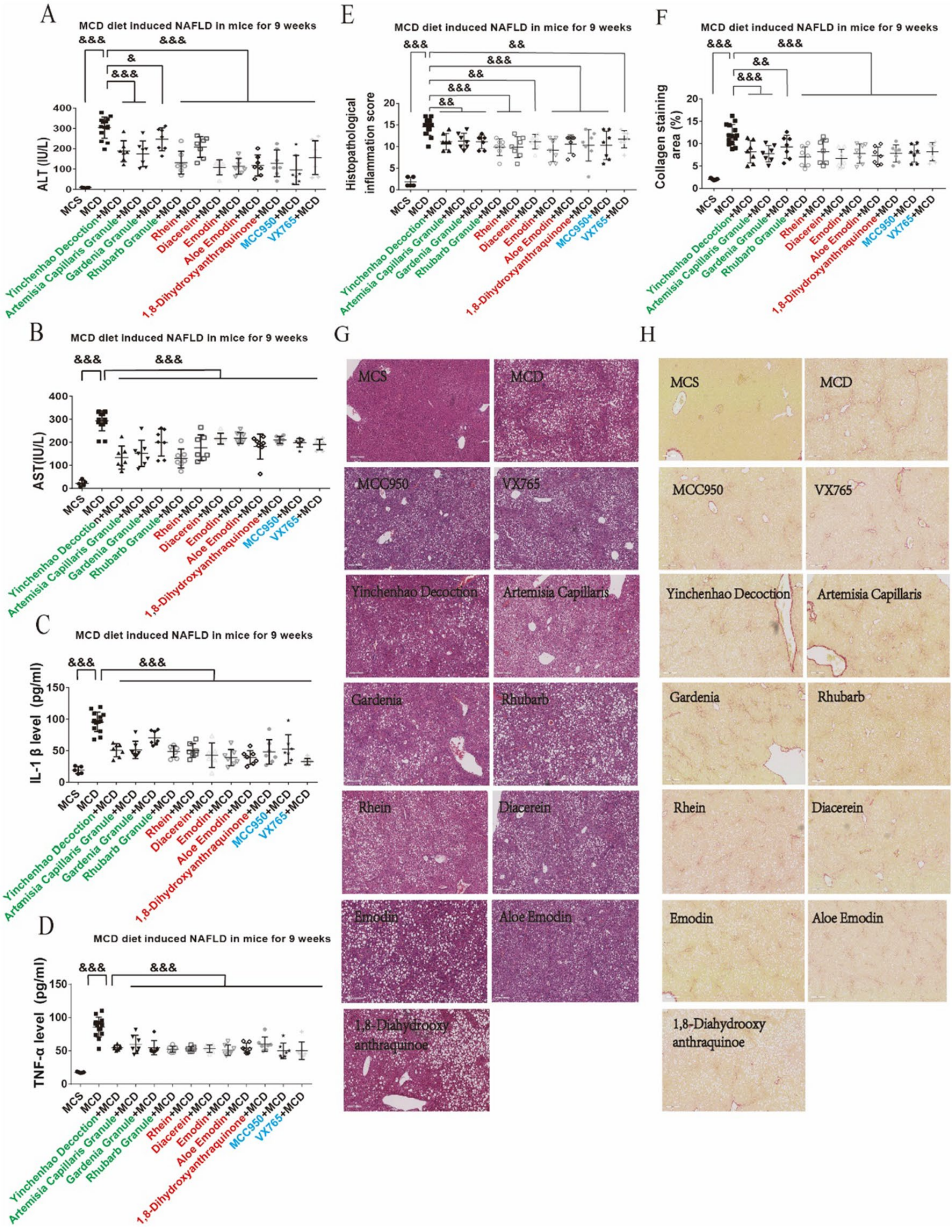
RFAs improved NAFLD by inhibiting NLRP3 inflammasome. C57 BL/6 mice were fed by MCD diet for 9 weeks. RFAs (rhein/diacerein, emodin, aloe emodin, 1,8-dihydroxyanthraquinone), herbs in yichenhao decoction and yichenhao decoction as well as inflammasome inhibitors MCC950 and VX765 were given by gavage every 2 days when MCD diet treatment started. Serum ALT (**A**) and AST (**B**) were detected by ALT/AST kits, serum IL-1β (**C**) and TNF-α (**D**) were detected by ELISA. Histopathological inflammation score was accessed (**E**) and H&E stain were also detected (**G**). Collagen staining was detected semi quantitatively (**F**) and sirius red staining were detected (**H**). Control group was fed by MCS diet (n = 5/group); Model group was fed by MCD diet (n = 14/group); Intervention groups were fed by MCD diet and different drugs (n = 7/group). For multiple comparisons, one-way ANOVA coupled with LSD’s post hoc testing was performed. &: p < 0.05; &&: p < 0.01; &&&: p < 0.001. ALT, alanine aminotransferase; ANOVA, analysis of variance; AST, aspartate aminotransferase; ELISA, enzyme linked immunosorbent assay; H&E, hematoxylin and eosin; IL-1β, interleukin-1 beta; MCD, methionine and choline deficiency; MCS, methionine-choline-supplemented; TNF-α, tumor necrosis factor-alpha

Regulation of NLRP3 inflammasome improved hepatic inflammation and fibrosis in NAFLD [33]. The role of these effective RFAs, rhubarb and YCHD in improving MCD diet induced mice NAFLD by inhibiting NLRP3 inflammasome was explored. NAFLD mice established by MCD diet for 9 weeks were administered by these effective RFAs, rhubarb and YCHD as well as inflammasome inhibitors MCC950 [34] and VX765 [35]. All these drugs improved liver function including ALT (Fig. 4A) and AST (Fig. 4B), serum inflammation such as IL-1β (Fig. 4C) and TNF-α (Fig. 4D) as well as histopathological inflammation score (Fig. 4E) according to Knodell scoring system [36] and collagen deposition (Fig. 4F). Similar results were also showed in NAFLD mice established by MCD diet for 3 weeks that all these drugs improved liver function including ALT (Additional file 1: Fig. S9A) and AST (Additional file 1: Fig. S9B) as well as serum inflammation such as IL-1β (Additional file 1: Fig. S9C) and TNF-α (Additional file 1: Fig. S9D). These effective RFAs also improved some main pathological changes including fat deposition and steatosis of hepatocytes (Additional file 1: Fig. S9E, Fig. 4G). Sirius red staining showed rhubarb and RFAs and inflammasome inhibitors had significantly improved liver fibrosis: the fibrous septa of liver tissue in the model group were connected with each other to form pseudolobules, while these effective RFAs, rhubarb and YCHD inhibited the interconnection between fibrous septa and the formation of pseudolobules (Fig. 4H).

## Discussion

### RFAs as NLRP3 inflammasome blockers

On the one hand, NLRP3 inflammasome activation characterized by the cleavage of pro-caspase-1 results in the cleavage and the secretion of IL-1 family such as IL-1 and IL-18 [5, 12]. On the other hand, macrophages activated by LPS can induce membranous TNF-α, while the cleavage of membranous TNF-α is just regulated by a disintegrin and metalloproteases (ADAMs) [13, 14]. Therefore, differential expression of IL-1β and TNF-α can identify the drugs to target NLRP3 inflammasome. RFAs including rhein/diacerein, emodin, aloe emodin and 1,8-dihydroxyanthraquinone inhibited NLRP3 inflammasome by inhibiting IL-1β, but had no effect on TNF-α. Besides these effective RFAs inhibited not only the cleavage of pro-caspase-1 and but also the formation of ASC specks which is regarded as one of the most important characteristics of NLRP3 inflammasome [37]. These results also supported the role of RFAs in regulating NLRP3 inflammasome.

YCHD showed potential role in treating many liver diseases, such as hepatitis, liver fibrosis and liver cirrhosis [24], and NLRP3 inflammasome takes the vital role in the process of hepatitis, liver fibrosis and liver cirrhosis in different liver diseases including NAFLD, Alcoholic fatty liver disease (ALD) and viral hepatitis [38, 39]. Furthermore, RFAs are the vital components of herd rhubarb and rhubarb is one of the most important herds involved in YCHD [22, 24]. Therefore, we suppose NLRP3 inflammasome probably is one of the most important targets of YCHD to treat liver diseases and RFAs from rhubarb are the main components of YCHD to make the vital role in inhibiting NLRP3 inflammasome and the results also confirmed our hypothesis.

### Rhubarb and RFAs inhibited the transcription and assembly of NLRP3

On the one hand, these effective RFAs inhibited the expression of NLRP3 protein and mRNA in a dose-dependent manner, indicating that RFAs inhibited the transcription of NLRP3. On the other hand, LPS binds to macrophage TLR4 and then activates the phosphorylation of TLR4 signaling pathway signaling proteins such as P65 in NF-κ B pathway and p38, ERK and JNK in MAPK signaling pathway [30, 31], while these effective RFAs had no effect on the phosphorylation of p65, p38, ERK and JNK. Therefore, RFAs directly regulate the transcription of NLRP3 independent of TLR4 signaling pathway.

The cascade reaction of NLRP3, ASC and pro-caspase-1 leads to the cleavage of pro-caspase-1, which further leads to the cleavage of IL-1 family and GSDMD which contribute to inflammation and pyroptosis [40, 41]. In order to study the effect of effective RFAs on NLRP3 assembly, cell culture medium with LPS was changed and then mouse BMDMs were pretreated with the abovementioned anthraquinones and stimulated by nigericin. Effective RFAs inhibited cleavage of pro-caspase-1, the formation of ASC specks and the secretion of IL-1β, but had no effect on NLRP3 protein expression. These results predicted the role of these effective RFAs in regulating NLRP3 assembly. Besides, these effective RFAs directly inhibited the binding between NLRP3 and ASC. This result furthermore indicated that effective RFAs inhibited the assembly of NLRP3.

### RFAs improved NAFLD by regulating NLRP3 inflammasome

NLRP3 inflammasome induced by metabolic stress in NAFLD can mediate pyrosis and inflammation which promote the progress of NAFLD [38, 42]. On the one hand, a variety of risk signals in the process of NAFLD such as endotoxin produced by intestinal flora migration, oxidized lipids and damage associated molecular patterns (DAMPs) can directly lead to the expression of NLRP3 in liver tissue through TLR4 [32]. Although the ligand of NLRP3 and the initial activation mechanism of NLRP3 inflammasome are still unknown, NLRP3 can be activated by sensing the stress stimulation of different metabolites and the risk signals generated in hepatocyte injury and death, for example, cholesterol can directly induce the activation of NLRP3 inflammasome [10, 43, 44]. In mouse BMDMs, RFAs including rhein, diacerein, emodin, aloe emodin, and 1,8-dihydroxyanthraquinone, rhubarb and YCHD just inhibited IL-1β, but had no effect on TNF-α. Similar results were also showed in mouse Kuffer cells and primary hepatocytes. Besides, these effective RFAs not only inhibited the formation of ASC in mouse Kuffer cells, but also inhibited the cleavage of pro-caspase-1 in mouse primary hepatocytes. These results showed rhubarb and RFAs could inhibit the metabolic stress induced NLRP3 inflammasome.

DAMPs released from injurious hepatocytes produced inflammatory factors by activating Kuffer cells, so as to further promoted and maintained the activation of hepatic stellate cells (HSCs), and finally lead to the continuous secretion of collagen and fibers by HSCs, thus forming hepatic fibrosis and even cirrhosis [45]. Inflammasome not only participate in hepatocyte injury, but also participate in the process of activation and release of inflammatory factors by Kuffer cells which directly promote the production of collagen and fibers by HSCs [33, 46]. These effective RFAs not noly inhibited the NLRP3 activation in mouse hepatocytes and Kuffer cells, but also improved mice NAFLD. RFAs including rhein, diacerein, emodin, aloe emodin, and 1,8-dihydroxyanthraquinone, rhubarb and YCHD as well as inflammasome inhibitors improved liver function including ALT and AST as well as serum IL-1β and TNF-α. Furthermore, H&E staining and Sirius red staining results showed those drugs not only improved histopathological inflammation score but also liver fibrosis. These results predicted that RFAs including rhein, diacerein, emodin, aloe emodin, and 1,8-dihydroxyanthraquinone could be regarded as NLRP3 inflammasome blockers to treat NAFLD.

## Conclusion

RFAs including rhein/diacerein, emodin, aloe emodin and 1,8-dihydroxyanthraquinone inhibited NLRP3 inflammasome. RFAs inhibited not only the expression of NLRP3 but also the NLRP3 inflammasome assembly. RFAs including rhein/diacerein, emodin, aloe emodin and 1,8-dihydroxyanthraquinone improved NAFLD by inhibiting NLRP3 inflammasome. Rhubarb and YCHD also inhibited NLRP3 inflammasome and improved NAFLD. RFAs are the potential NLRP3 inflammasome blockers for treating NAFLD clinically. NLRP3 inflammasome also are important therapeutic target of rhubarb and YCHD.

Although RFAs includig rhein, diacerein, emodin, aloe emodin, and 1,8-dihydroxyanthraquinone were screened out as NLRP3 inflammasome blockers to treat NAFLD, while many questions still exit in this paper and we will try to solve these problems in our next researches: (1) there is no clinical trial to provide more evidence for the role of RFAs in treating NAFLD; (2) the deeper mechanism that RFAs inhibited NLRP3 inflammasome are still unkown; (3) How to choose the clinical application of these effective RFAs or rhubarb or prescription containing rhubarb in clinical treatment of NAFLD? All these existing problems will inspire us to make greater efforts to solve them in our future work.

## Chemical compounds

Acrylamide (PubChem CID: 6579), Aluminum potassium sulfate (PubChem CID: 24856), Ammonium persulfate (PubChem CID: 62648), Calcium pyrophosphate (PubChem CID: 24632), Chloroform (PubChem CID: 6212), Cholesterol (PubChem CID: 5997), Dimethyl sulfoxide (PubChem CID: 679), Disodium hydrogen phosphate (PubChem CID: 24203), Ethanol (PubChem CID: 702), Formaldehyde (PubChem CID: 712), Glycine (PubChem CID: 750), Methanol (PubChem CID: 887), Nigericin (PubChem CID: 34230), Potassium chloride (PubChem CID: 4873), Potassium dihydrogen phosphate (PubChem CID: 516951), Silicon dioxide (PubChem CID: 24261), Sodium chloride (PubChem CID: 5234), Sodium dihydrogen phosphate (PubChem CID: 23672064), Sodium dodecyl sulfate (PubChem CID: 3423265), Tris (PubChem CID: 6503), Uric acid (PubChem CID: 1175), Xylene (PubChem CID: 6850715).

## Supplementary Information

**Additional file 1. Figure S1.** RFAs inhibited NLRP3 inflammasome induced by uric acid, alum, SiO2 and CPPD. **Figure S2.** RFAs inhibited NLRP3 inflammasome in a dose dependent manner. **Figure S3.** RFAs inhibited the transcription of NLRP3 in a dose dependent manner. **Figure S4.** RFAs inhibited the assembly of NLRP3 inflammasome in a dose dependent manner. **Figure S5.** RFAs inhibited cholesterol induced NLRP3 inflammasome in mouse BMDMs in a dose dependent manner. **Figure S6.** RFAs inhibited cholesterol induced NLRP3 inflammasome in mouse Kuffer cells. **Figure S7.** RFAs inhibited cholesterol induced NLRP3 inflammasome in mouse primary hepatocytes. **Figure S8.** RFAs inhibited ALT and AST in mouse primary hepatocytes stimulated by LPS + cholesterol. **Figure S9.** RFAs improved MCD diet induced mice NAFLD by inhibiting NLRP3 inflammasome in the third week. **Table S1.** Main materials.

## Abbreviations

ADAMs: A disintegrin and metalloproteases
ANOVA: Analysis of variance
ALT: Alanine aminotransferase
Alum: Aluminum potassium sulfate
ASC: Apoptosis-associated speck-like protein containing CARD
AST: Aspartate aminotransferase
ATP: Adenosine triphosphate
BMDMs: Bone marrow-derived macrophages, BSA, bovine serum albumin
CPPD: Calcium pyrophosphate
DAPI: 4',6-Diamidino-2-phenylindole
ELISA: Enzyme linked immunosorbent assay
GSDMD: Gasdermin D
H&E: Hematoxylin and eosin
HSCs: Hepatic stellate cells
IL-1β: Interleukin-1 beta
LDH: Lactate dehydrogenase
LPS: Lipopolysaccharide
MCD: Methionine and choline deficiency
MCS: Methionine-choline-supplemented
NAFLD: Nonalcoholic fatty liver disease
NASH: Nonalcoholic steatohepatitis
NS: No significance
RFAs: Rhubarb free anthraquinones
ROS: Reactive oxygen species
SiO_2_: Silicon dioxide
TLR4: Toll like receptor 4
TNF-α: Tumor necrosis factor-alpha
YCHD: Yinchenhao decoction
