# Supplementary material for: Rhubarb free anthraquinones improved mice nonalcoholic fatty liver disease by inhibiting NLRP3 inflammasome
Source: J Transl Med. 2022 Jun 28;20:294. doi: 10.1186/s12967-022-03495-4 (PMC9238089; doi:10.1186/s12967-022-03495-4)
Supplement: Supplementary file 1 — Additional file 1. Figure S1. RFAs inhibited NLRP3 inflammasome induced by uric acid, alum, SiO2 and CPPD. Figure S2. RFAs inhibited NLRP3 inflammasome in a dose dependent manner. Figure S3. RFAs inhibited the transcription of NLRP3 in a dose dependent manner. Figure S4. RFAs inhibited the assembly of NLRP3 inflammasome in a dose dependent manner. Figure S5. RFAs inhibited cholesterol induced NLRP3 inflammasome in mouse BMDMs in a dose dependent manner. Figure S6. RFAs inhibited cholesterol induced NLRP3 inflammasome in mouse Kuffer cells. Figure S7. RFAs inhibited cholesterol induced NLRP3 inflammasome in mouse primary hepatocytes. Figure S8. RFAs inhibited ALT and AST in mouse primary hepatocytes stimulated by LPS + cholesterol. Figure S9. RFAs improved MCD diet induced mice NAFLD by inhibiting NLRP3 inflammasome in the third week. Table S1. Main materials. [file 12967_2022_3495_MOESM1_ESM.docx]

# Supplementary Materials

## Supplementary figures


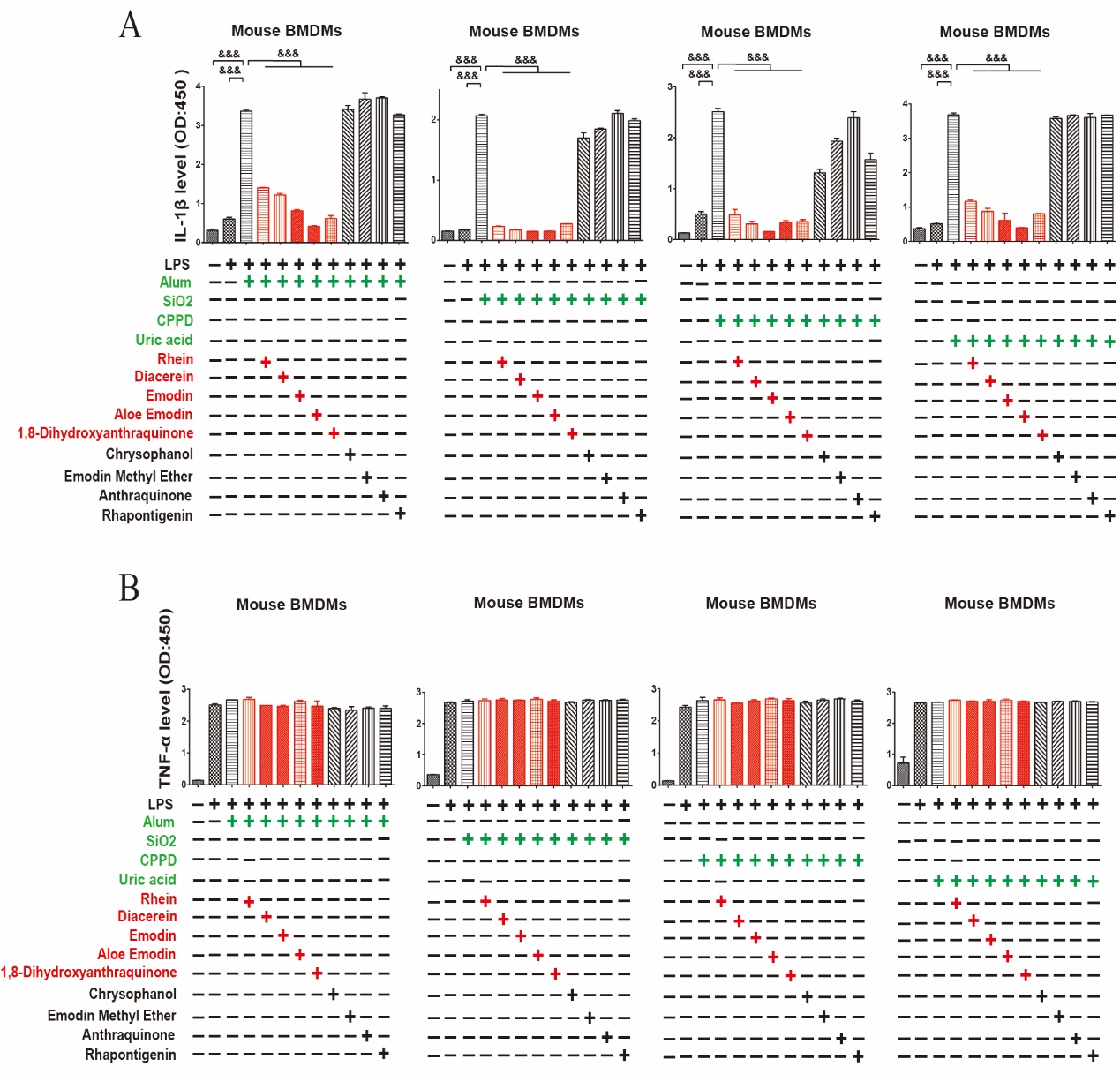


### Additional file 1: Fig S 1. RFAs inhibited NLRP3 inflammasome induced by uric acid, alum, SiO_2_ and CPPD.

Moue BMDMs were pretreated with RFAs (40μM rhein / 40μM diacerein, 40μM emodin, 40μM aloe emodin, 40μM 1,8-dihydroxyanthraquinone, 20μM chrysophanol, 4μM emodin methyl ether, 4μM anthraquinone) for 30 minutes and then stimulated with LPS (100ng / ml) for 4 hours. uric acid (100μg / ml), alum (100μg / ml), SiO_2_ (100μg / ml) and CPPD (100μg / ml) were added for 12 hours. IL-1β (**A**) and TNF-α (**B**) in cell culture supernatant were detected by ELISA (n=3 / group). Data are presented as mean ± SEM. For multiple comparisons, one-way ANOVA coupled with LSD’s post hoc testing was performed. &: P<0.05; &&: p<0.01; &&&: p<0.001. ANOVA, analysis of variance; BMDMs, bone marrow-derived macrophages; CPPD, calcium pyrophosphate; ELISA, enzyme linked immunosorbent assay; IL-1β, interleukin-1 beta; LPS, lipopolysaccharide; SiO_2_, silicon dioxide; TNF-α, tumor necrosis factor-alpha.


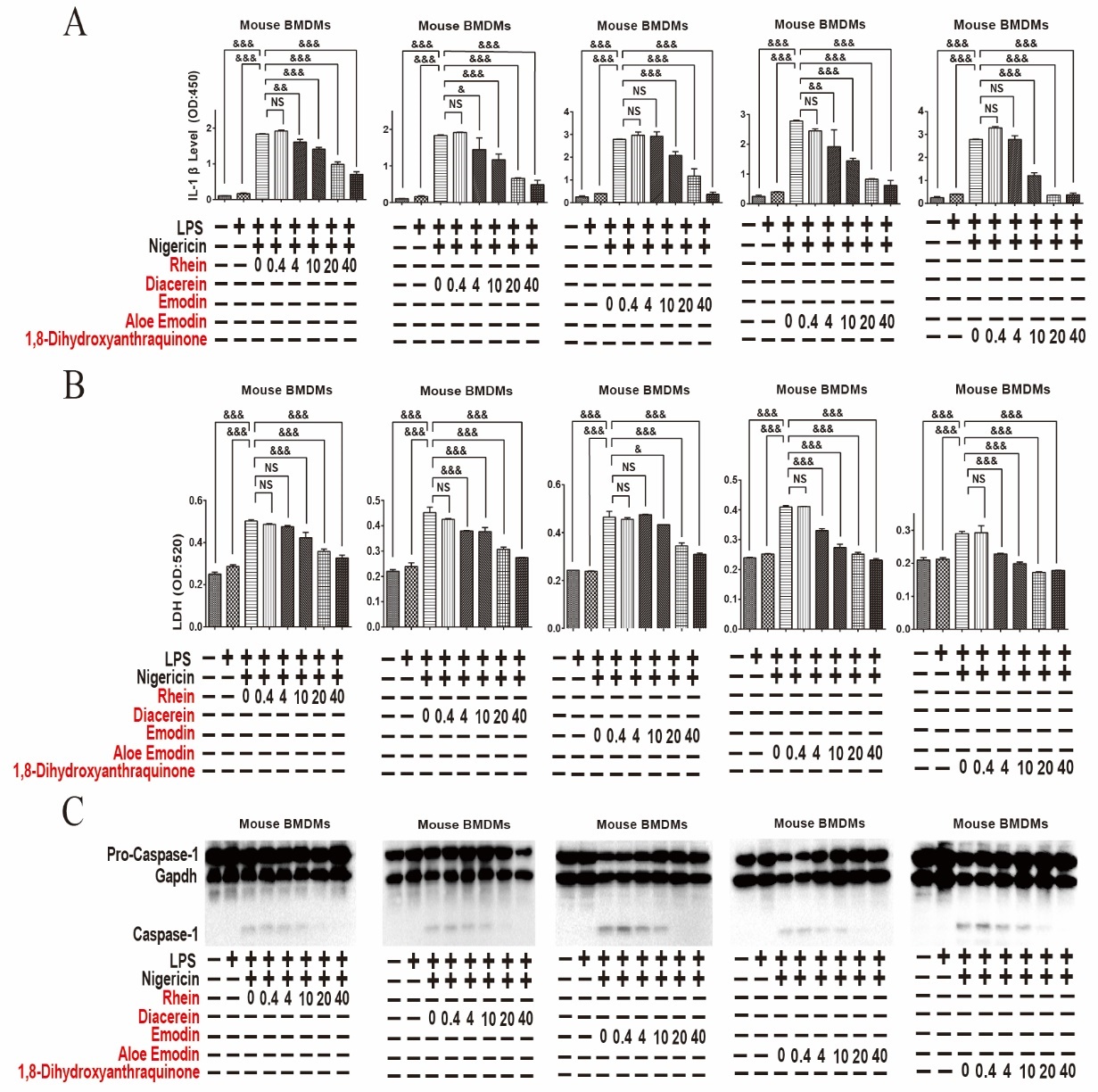


### Additional file 1: Fig S 2. RFAs inhibited NLRP3 inflammasome in a dose dependent manner.

Mouse BMDMs were pretreated by rhein, diacerein, emodin, aloe emodin and 1,8-dihydroxyanthraquinone with the concentration of 40μM, 20μM, 10μM, 4μM and 0.4μM for 30 minutes, and then stimulated with LPS (100ng / ml) for 4 hours. Nigericin (2.5μM) was added for 2 hours. IL-1β (**A**) in cell culture supernatant were detected by ELISA (n=3 / group), LDH (**B**) in cell culture supernatant were detected by biochemical kit (n=3 / group), The cleavage of pro-caspase-1 (**C**) was detected by western blot. Data are presented as mean ± SEM. For multiple comparisons, one-way ANOVA coupled with LSD’s post hoc testing was performed. &: P<0.05; &&: p<0.01; &&&: p<0.001. ANOVA, analysis of variance; BMDMs, bone marrow-derived macrophages; ELISA, enzyme linked immunosorbent assay; IL-1β, interleukin-1 beta; LDH, lactate dehydrogenase; LPS, lipopolysaccharide; NS, no significance; TNF-α, tumor necrosis factor-alpha.


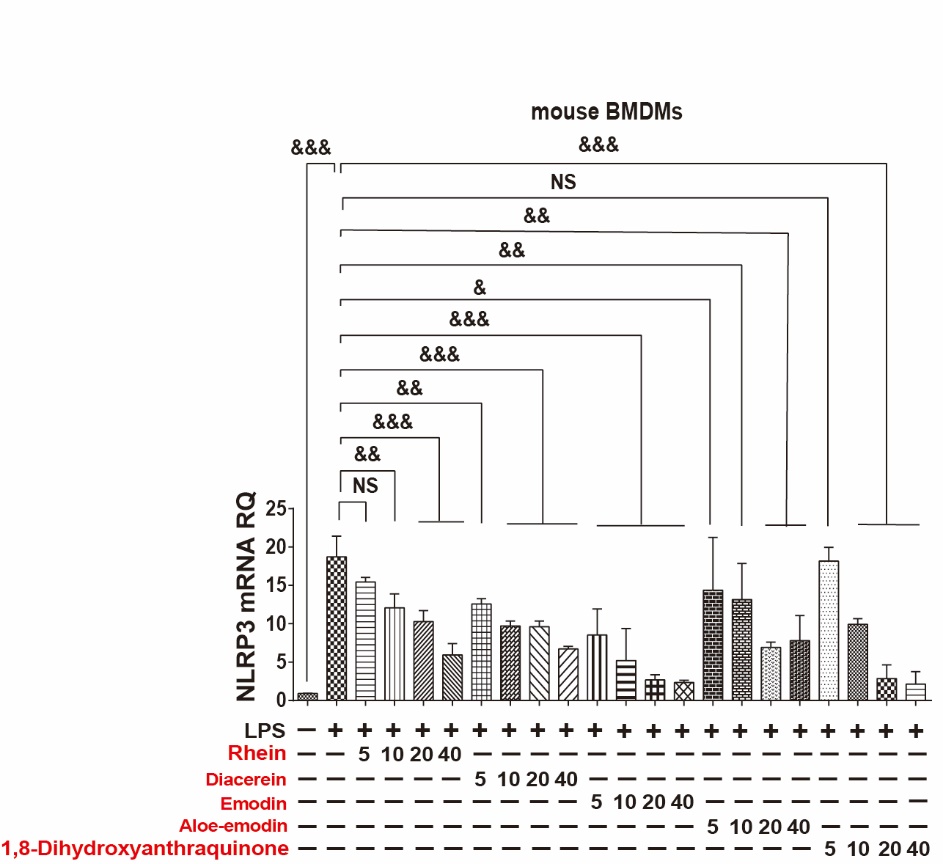


### Additional file 1: Fig S 3. RFAs inhibited the transcription of NLRP3 in a dose dependent manner.

Mouse BMDMs were pretreated by rhein, diacerein, emodin, aloe emodin and 1,8-dihydroxyanthraquinone with the concentration of 40μM, 20μM, 10μM and 5μM for 30min and then stimulated with 100ng / ml LPS for 1 hours. NLRP3 mRNA were detected by RT-qPCR (n=3 / group). Data are presented as mean ± SEM. For multiple comparisons, one-way ANOVA coupled with LSD’s post hoc testing was performed. &: P<0.05; &&: p<0.01; &&&: p<0.001. ANOVA, analysis of variance; BMDMs, bone marrow-derived macrophages; LPS, lipopolysaccharide; NS, no significance; RT-qPCR, quantitative reverse transcription polymerase chain reaction.


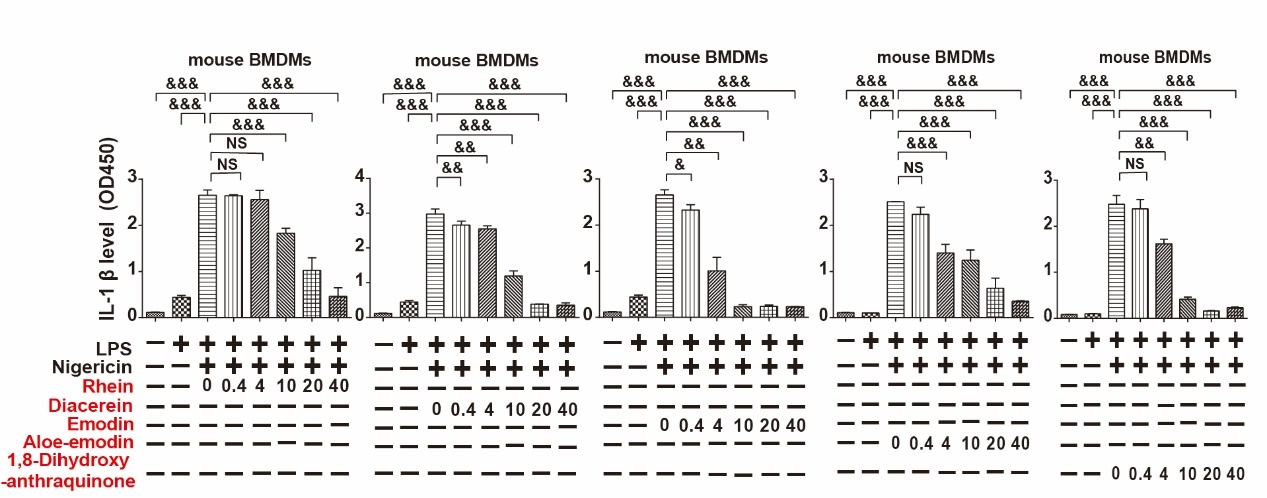


### Additional file 1: Fig S 4. RFAs inhibited the assembly of NLRP3 inflammasome in a dose dependent manner.

Mouse BMDMs were stimulated by LPS (100ng / ml) for 4 hours before the media was changed. Rhein, diacerein, emodin, aloe emodin and 1,8-dihydroxyanthraquinone with the concentrations of 40μM, 20μM, 10μM, 4μM and 0.4μM were added for 30 minutes and then stimulated with nigericin (2.5μM) for 2 hours. IL-1β in cell culture supernatant was detected by ELISA (n=3 / group). Data are presented as mean ± SEM. For multiple comparisons, one-way ANOVA coupled with LSD’s post hoc testing was performed. &: P<0.05; &&: p<0.01; &&&: p<0.001. ANOVA, analysis of variance; BMDMs, bone marrow-derived macrophages; ELISA, enzyme linked immunosorbent assay; IL-1β, interleukin-1 beta; LPS, lipopolysaccharide, NS, no significance.


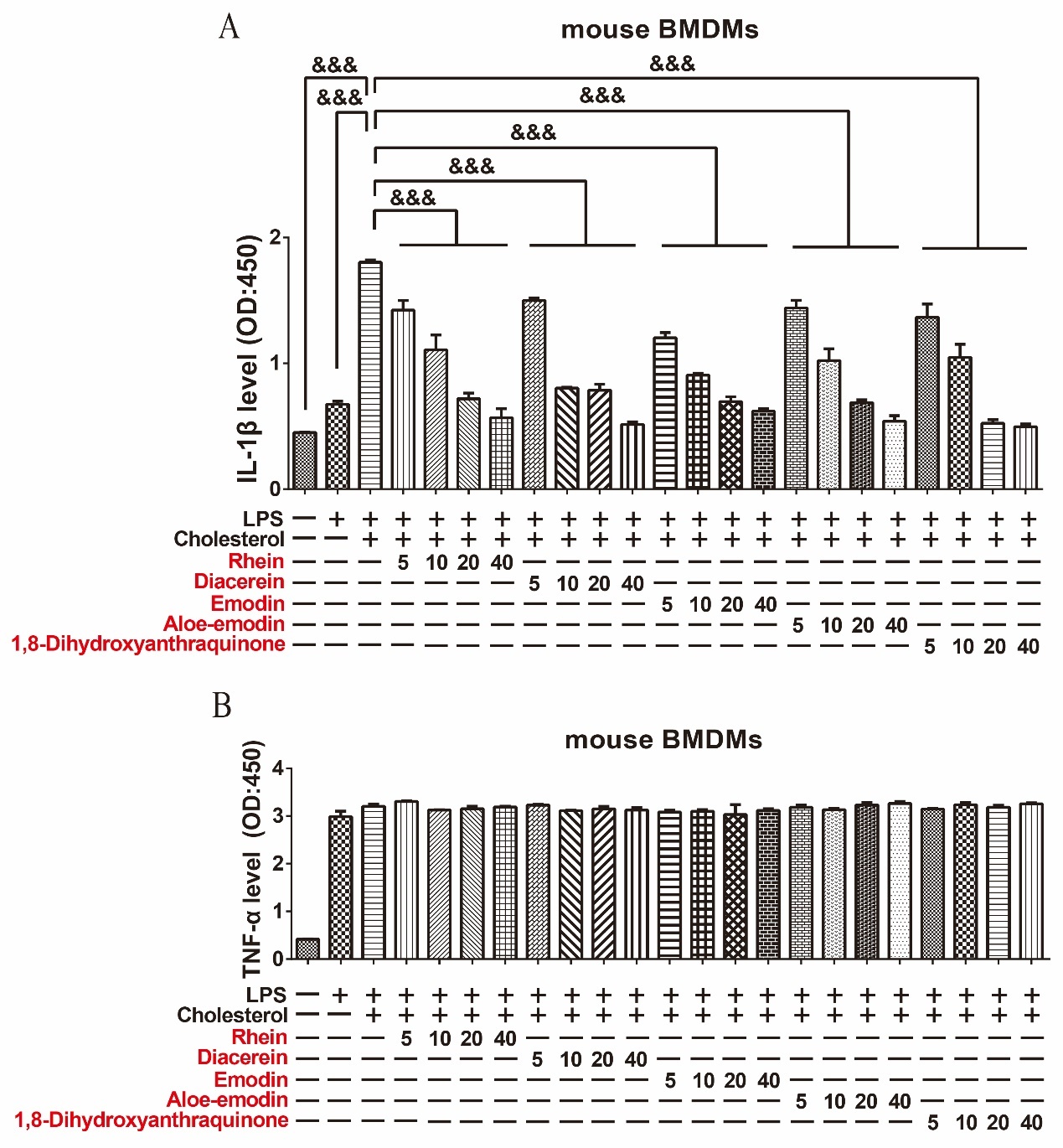


### Additional file 1: Fig S 5. RFAs inhibited cholesterol induced NLRP3 inflammasome in mouse BMDMs in a dose dependent manner.

Mouse BMDMs were pretreated by RFAs (Rhein, diacerein, emodin, aloe emodin and 1,8-dihydroxyanthraquinone with the concentrations of 40μM, 20μM, 10μM and 5μM) for 30 minutes and then stimulated with LPS (100ng/ml) for 4 hours and cholesterol (100μg / ml) for 12 hours. IL-1β (**A**) and TNF-α (**B**) in cell culture supernatant were detected by ELISA (n=3 / group). Data are presented as mean ± SEM. For multiple comparisons, one-way ANOVA coupled with LSD’s post hoc testing was performed. &: P<0.05; &&: p<0.01; &&&: p<0.001. ANOVA, analysis of variance; BMDMs, bone marrow-derived macrophages; ELISA, enzyme linked immunosorbent assay; IL-1β, interleukin-1 beta; LPS, lipopolysaccharide; TNF-α, tumor necrosis factor-alpha.


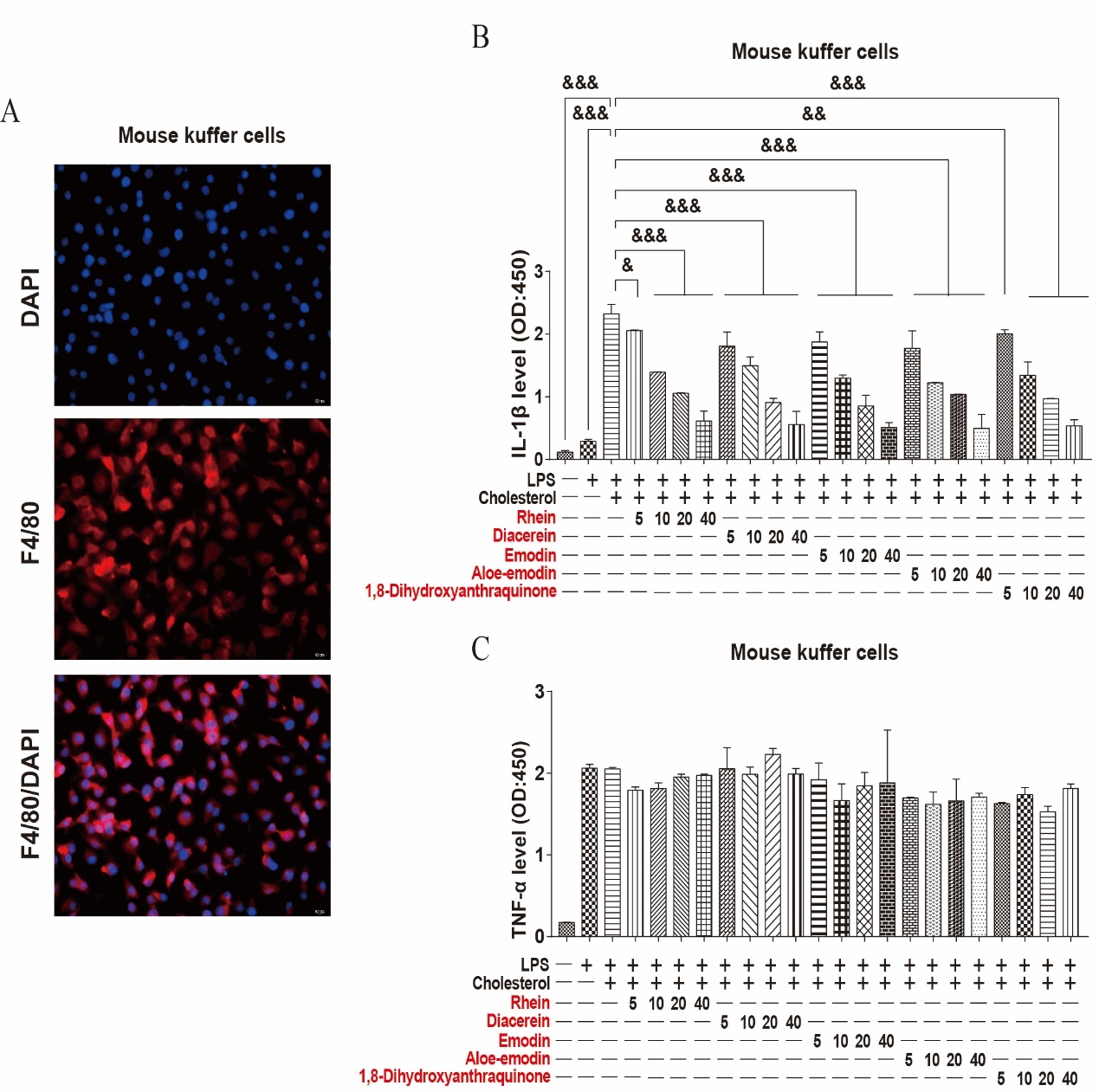


### Additional file 1: Fig S 6. RFAs inhibited cholesterol induced NLRP3 inflammasome in mouse Kuffer cells.

Mouse Kuffer cells were identified by immunofluorescence through detecting F4/80 (**A**). Mouse Kuffer cells were pretreated by RFAs (Rhein, diacerein, emodin, aloe emodin and 1,8-dihydroxyanthraquinone with the concentrations of 40μM, 20μM, 10μM and 5μM) for 30 minutes and then stimulated with LPS (100ng / ml) for 4 hours and cholesterol (100μg / ml) for 12 hours. IL-1β (**B**) and TNF-α (**C**) in cell culture supernatant were detected by ELISA (n=3 / group). Data are presented as mean ± SEM. For multiple comparisons, one-way ANOVA coupled with LSD’s post hoc testing was performed. &: P<0.05; &&: p<0.01; &&&: p<0.001. ANOVA, analysis of variance; DAPI, 4',6-diamidino-2-phenylindole; ELISA, enzyme linked immunosorbent assay; IL-1β, interleukin-1 beta; LPS, lipopolysaccharide; TNF-α, tumor necrosis factor-alpha.


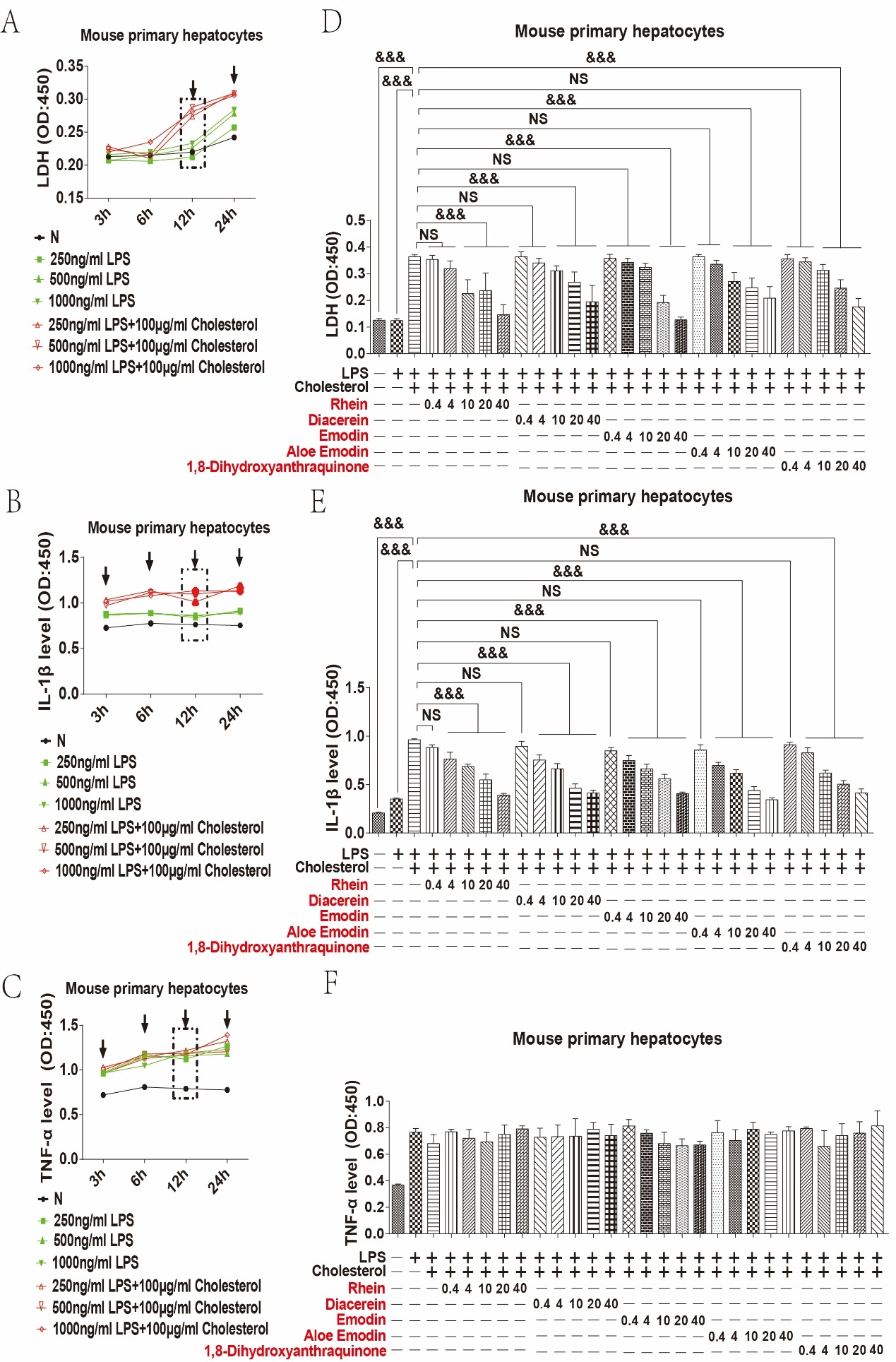


### Additional file 1: Fig S 7. RFAs inhibited cholesterol induced NLRP3 inflammasome in mouse primary hepatocytes.

Mouse primary hepatocytes were stimulated by LPS (250ng / ml, 500ng / ml and 1000ng / ml) for 4 hours and then stimulated by cholesterol (100μg / ml) for 12 hours, LDH (**A**) were detected by LDH kits (n=1 / group); IL-1β (**B**) and TNF-α (**C**) were detected by ELISA (n=1 / group). Mouse primary hepatocytes were pretreated by RFAs (Rhein, diacerein, emodin, aloe emodin and 1,8-dihydroxyanthraquinone with the concentrations of 40μM, 20μM, 10μM and 5μM) for 30 minutes and then stimulated with LPS (1000ng / ml) for 4 hours and cholesterol (100μg / ml) for 12 hours. LDH were detected by LDH kits (**D**) (n=3 / group); IL-1β (**E**) and TNF-α (**F**) were detected by ELISA (n=3 / group). Data are presented as mean ± SEM. For multiple comparisons, one-way ANOVA coupled with LSD’s post hoc testing was performed. &: P<0.05; &&: p<0.01; &&&: p<0.001. ANOVA, analysis of variance; ELISA, enzyme linked immunosorbent assay; IL-1β, interleukin-1 beta; LDH, lactate dehydrogenase; LPS, lipopolysaccharide; NS, no significance; TNF-α, tumor necrosis factor-alpha.


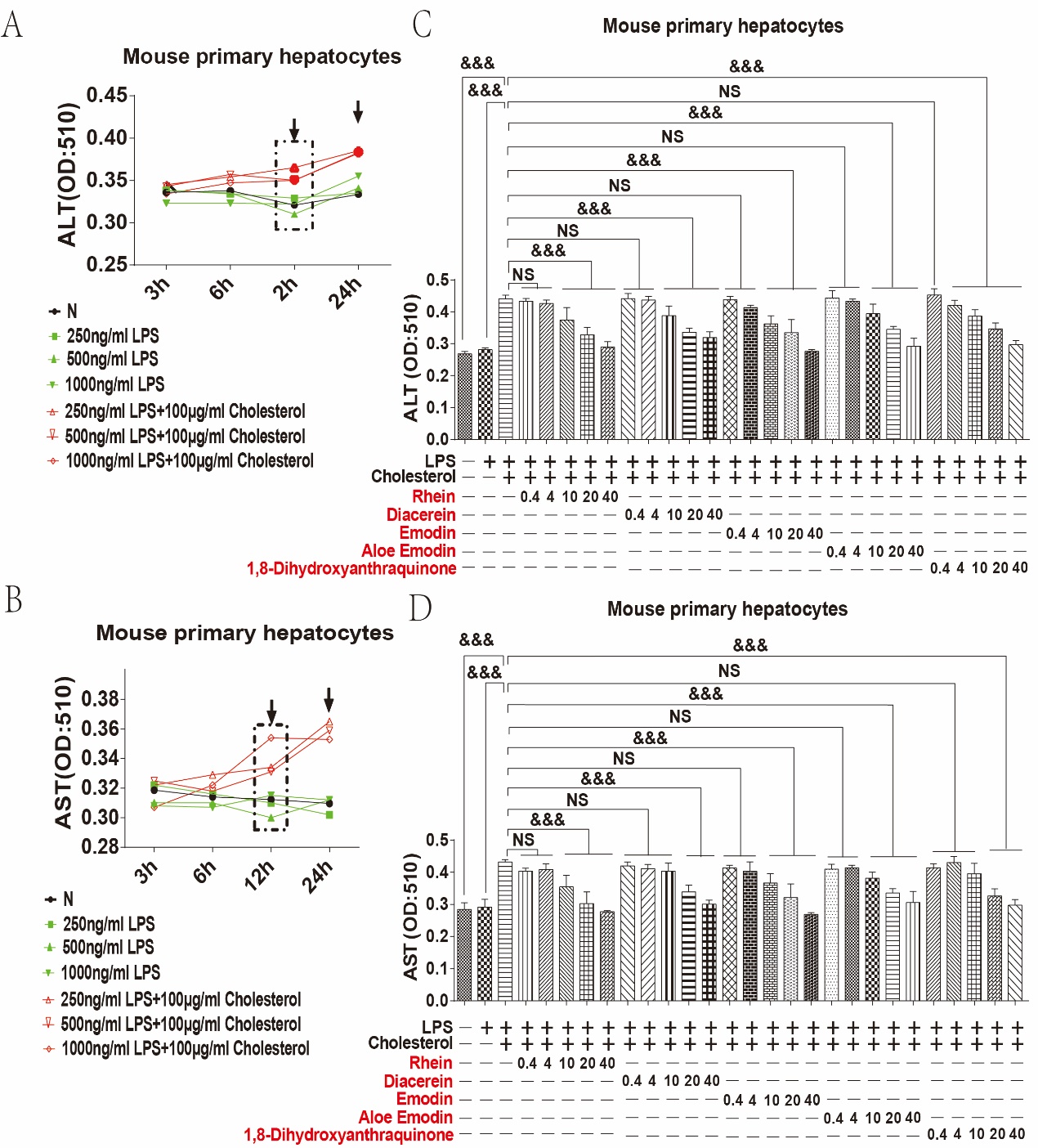


### Additional file 1: Fig S 8. RFAs inhibited ALT and AST in mouse primary hepatocytes stimulated by LPS + cholesterol.

Mouse primary hepatocytes were stimulated by LPS (250ng / ml, 500ng / ml and 1000ng / ml) for 4 hours and then stimulated by cholesterol (100μg / ml) for 12 hours, ALT (**A**) and AST (**B**) were detected by ALT / AST kits (n=1 / group). Mouse primary hepatocytes were pretreated by RFAs (Rhein, diacerein, emodin, aloe emodin and 1,8-dihydroxyanthraquinone with the concentrations of 40μM, 20μM, 10μM and 5μM) for 30 minutes and then stimulated with LPS (1000ng / ml) for 4 hours and cholesterol (100μg / ml) for 12 hours. ALT (**C**) and AST (**D**) were detected by ALT / AST kits (n=3 / group). Data are presented as mean ± SEM. For multiple comparisons, one-way ANOVA coupled with LSD’s post hoc testing was performed. &: P<0.05; &&: p<0.01; &&&: p<0.001. ALT, alanine aminotransferase; ANOVA, analysis of variance; AST, aspartate aminotransferase; ELISA, enzyme linked immunosorbent assay; IL-1β, interleukin-1 beta; LDH, lactate dehydrogenase; LPS, lipopolysaccharide; NS, no significance; TNF-α, tumor necrosis factor-alpha.


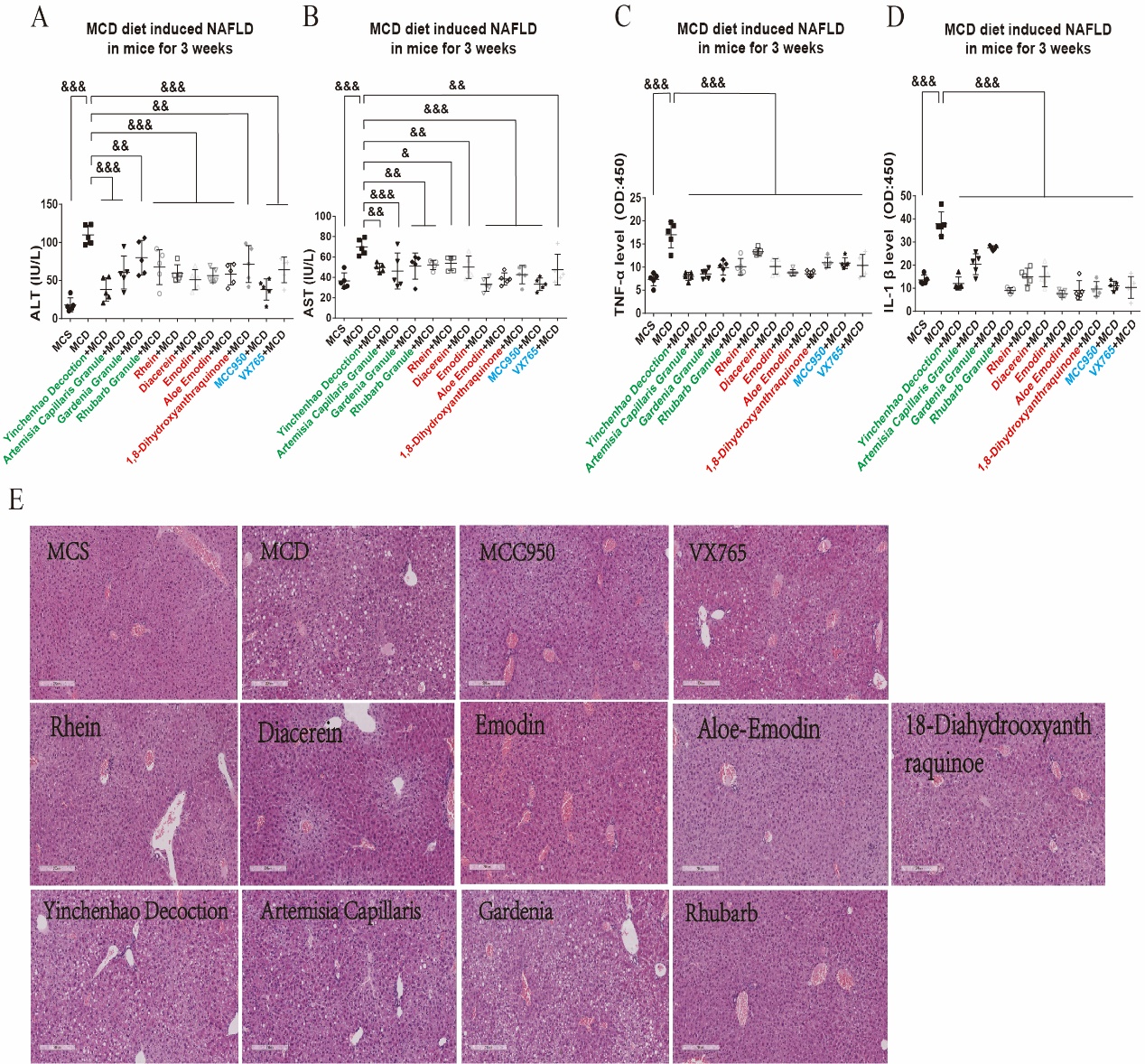


### Additional file 1: Fig S 9. RFAs improved MCD diet induced mice NAFLD by inhibiting NLRP3 inflammasome in the third week.

C57 BL/6 mice were fed by MCD diet for 3 weeks. RFAs (rhein / diacerein, emodin, aloe emodin, 1,8-dihydroxyanthraquinone), rhubarb and yinchenhao decoction as well as inflammasome inhibitors MCC950 and VX765 were given by gavage every 2 days when MCD diet treatment started. Serum ALT (**A**) and AST (**B**) were detected by ALT / AST kit, serum IL-1β (**C**) and TNF-α (**D**) were detected by ELISA. H&E stain and sirius red staining were also detected (**E**). Control group was fed by MCS diet (n=5 / group); Model group was fed by MCD diet (n=5 / group); Intervention groups were fed by MCD diet and different drugs (n=5 / group). For multiple comparisons, one-way ANOVA coupled with LSD’s post hoc testing was performed. &: P<0.05; &&: p<0.01; &&&: p<0.001. ALT, alanine aminotransferase; ANOVA, analysis of variance; AST, aspartate aminotransferase; ELISA, enzyme linked immunosorbent assay; H&E, hematoxylin and eosin; IL-1β, interleukin-1 beta; MCD, methionine and choline deficiency; MCS, methionine-choline-supplemented; TNF-α, tumor necrosis factor-alpha.

## Additional table

### Table S1 Main materials

| **REAGENT** | **SOURCE** | **IDENTIFIER** |
| --- | --- | --- |
| **Biochemical reagents & solutions** | | |
| Alum | Invivogen | 21645-51-2 |
| BSA | Sengon biotech | ST025 |
| CPPD | Sengon biotech | 7790-76-3 |
| DAPI | Beyotime | C1002 |
| LPS | Sigma | L2630 |
| Nigericin | Sigma | 481990 |
| SiO2 | Sengon biotech | 238-878-4 |
| 4% paraformaldehyde fix solution | Sengon biotech | E672002 |
| Lipofectamine 2000 | Invitrogen | 2030864 |
| Opti-MEM | Gibico | 2048098 |
| Protein A+G agrarose | Beyotime | P2055 |
| **Durgs** | | |
| Rhubarb | Tianjiang Pharmaceutical Co., Ltd | 18037004 |
| Rhein (Rhubarb) | Ronghe Pharmaceutical Technology Development Co., Ltd | 171019/171109 |
| Aloe Emodin (Rhubarb) | Ronghe Pharmaceutical Technology Development Co., Ltd | 190307/171216 |
| Anthraquinone (Rhubarb) | Ronghe Pharmaceutical Technology Development Co., Ltd | 170820 |
| Chrysophanol (Rhubarb) | Ronghe Pharmaceutical Technology Development Co., Ltd | 180309 |
| Diacerin (Rhubarb) | Ronghe Pharmaceutical Technology Development Co., Ltd | 171107/171224 |
| Emodin (Rhubarb) | Ronghe Pharmaceutical Technology Development Co., Ltd | 190120/180106 |
| Emodin methyl ether (Rhubarb) | Ronghe Pharmaceutical Technology Development Co., Ltd | 180905 |
| 1,8-dihydroxyanthraquinone (Rhubarb) | Ronghe Pharmaceutical Technology Development Co., Ltd | 170716/180325 |
| Artemisia Capillaris | Tianjiang Pharmaceutical Co., Ltd | 18086954 |
| Artemisinin (Artemisia Capillaris) | Ronghe Pharmaceutical Technology Development Co., Ltd | 190420 |
| Caffeic acid (Artemisia Capillaris) | Ronghe Pharmaceutical Technology Development Co., Ltd | 190425 |
| Chlorogenic acid (Artemisia Capillaris) | Ronghe Pharmaceutical Technology Development Co., Ltd | 190327 |
| Crocin I (Artemisia Capillaris) | Ronghe Pharmaceutical Technology Development Co., Ltd | 190124 |
| Crocin II (Artemisia Capillaris) | Ronghe Pharmaceutical Technology Development Co., Ltd | 190305 |
| Ferulic acid (Artemisia Capillaris) | Ronghe Pharmaceutical Technology Development Co., Ltd | 181109 |
| Hyperoside (Artemisia Capillaris) | Ronghe Pharmaceutical Technology Development Co., Ltd | 190402 |
| Quercetin (Artemisia Capillaris) | Ronghe Pharmaceutical Technology Development Co., Ltd | 190331 |
| Scopolactone (Artemisia Capillaris) | Ronghe Pharmaceutical Technology Development Co., Ltd | 190316 |
| 6-hydroxy-7-methoxycoumarin (Artemisia Capillaris) | Ronghe Pharmaceutical Technology Development Co., Ltd | 190103 |
| Gardenia | Tianjiang Pharmaceutical Co., Ltd | 1807664 |
| Genipin (Gardenia) | Ronghe Pharmaceutical Technology Development Co., Ltd | 181229 |
| Geniposide (Gardenia) | Ronghe Pharmaceutical Technology Development Co., Ltd | 181205 |
| Geniposidic acid (Gardenia) | Ronghe Pharmaceutical Technology Development Co., Ltd | 181227 |
| Genipin-1-β-gentibioside (Gardenia) | Ronghe Pharmaceutical Technology Development Co., Ltd | 190223 |
| **Inhibitors:** | | |
| MCC950 | Selleck Biotechnology Co., Ltd | S8930 |
| VX765 | Selleck Biotechnology Co., Ltd | S2228 |
| **RNA isolation, reverse and amplification kits** | | |
| RNAfast200 kit | Fastagen | 220011 |
| Reverse transcriptionand kit | TAKARA | RR036 |
| Amplification kit | Toyobo | 857100 |
| **Primers** | | |
| Mouse β-actin primers | Genewiz | N/S |
| Mouse IL-1β primers | Genewiz | N/S |
| Mouse TNF-α primers | Genewiz | N/S |
| Mouse NLRP3 primers | Genewiz | N/S |
| **Antibody** | | |
| p-P65/P65 | CST | 3033/6956 |
| p-ERK/ERK | CST | 4730/4695 |
| p-JNK/JNK | CST | 4668/9252 |
| p-P38/P38 | CST | 4511/9212 |
| NLRP3 | Adipogen | AG-20B-0014 |
| Caspase-1 | Adipogen | AG-20B-0048 |
| ASC | CST | 67824 |
| GAPDH | Proteinteck | 60004-l-lg |
| β-actin | Proteinteck | 66009-l-lg |
| β-actin | CST | 4970 |
| α-tubulin | Proteinteck | 66031-l-lg |
| Anti-rabbit IgG-Cy3 | Abcam | ab6939 |
| **Critical commercial Assays** | | |
| ALT kit | Nanjing Jiancheng bioengineering institute | C009-2-1 |
| AST kit | Nanjing Jiancheng bioengineering institute | C010-2-1 |
| LDH kit | Nanjing Jiancheng bioengineering institute | A020-2-2 |
| IL-1β ELISA kits | Thermo Fisher | 88-7013 |
| TNF-α ELISA kits | Thermo Fisher | 88-7324 |
